# Multi-omic characterization of microbial dynamics during spontaneous fermentation of sweet wine Picolit variety

**DOI:** 10.3389/fmicb.2026.1857803

**Published:** 2026-07-29

**Authors:** Michela Dell’Alma, Mattia Cesana, Pilar Kenny, Gregorio Peron, Cinzia Cafarella, Francesca Rigano, Luigi Mondello, Nicola Mangieri, Simona Pizzi, Pasquale Russo, Diego Mora, Giorgio Gargari

**Affiliations:** 1 Department of Food, Environmental and Nutritional Sciences, University of Milan, Milan, Italy; 2 Department of Molecular and Translational Medicine, University of Brescia, Brescia, Italy; 3 Department of Chemical, Biological, Pharmaceutical and Environmental Sciences, Messina Institute of Technology, University of Messina, Messina, Italy; 4 Department of Chemical, Biological, Pharmaceutical and Environmental Sciences, Chromaleont s.r.l., University of Messina, Messina, Italy

**Keywords:** fermentation ecology, metabolomics, metagenomics, non-*Saccharomyces* yeasts, spontaneous fermentation, wine microbiome

## Abstract

**Introduction:**

Spontaneous wine fermentation is driven by the ecological succession of vineyard-derived microorganisms, yet little is known about how this process unfolds in Picolit, a grape variety characterized by acinellatura (berry millerandage) and elevated sugar concentration. This study aimed to characterize the microbial and metabolic dynamics of spontaneous Picolit fermentation and to identify the ecological and functional transitions occurring throughout the process.

**Methods:**

An integrated multi-omic approach combining shotgun metagenomics and untargeted metabolomics was applied to spontaneous fermentations of Picolit grapes produced at Aquila del Torre, an organic and biodynamic winery located in Savorgnano del Torre (Friuli-Venezia Giulia, Italy), within the newly established “Friuli Colli Orientali Sottozona Savorgnano D.O.C.” Five fermentation stages were sampled and analyzed to investigate microbial succession, functional pathways and metabolomic changes.

**Results:**

The initial must displayed high microbial richness dominated by non-*Saccharomyces* yeasts, oxidative bacteria and *Botrytis cinerea*. An atypical persistence and increasing abundance of *B. cinerea* suggested a strong interaction between grape physiology and fungal activity. Early fermentation stages were characterized by diverse non-*Saccharomyces* taxa, including *Lachancea*, *Pichia*, *Torulaspora* and *Schizosaccharomyces*, which were associated with acid modulation, aromatic precursor release and phenolic turnover. From mid-fermentation onward, a multi-species *Saccharomyces* consortium established functional dominance, coinciding with a marked reduction in bacterial diversity and a transition from aroma-related metabolic pathways to stress adaptation functions. Multi-omic network analyses revealed a progressive loss of modularity as fermentation progressed and the system became more stable.

**Discussion:**

These findings demonstrate that spontaneous Picolit fermentation follows a distinctive ecological trajectory shaped by grape physiology, terroir and native microbial diversity. The persistence of *B. cinerea*, together with the succession of non-*Saccharomyces* and *Saccharomyces* populations, highlights unique microbial interactions that may contribute to wine identity. Overall, the results support the enological value of spontaneous fermentation and provide a microbial and functional framework for understanding and valorizing wines produced under the Savorgnano Bianco D.O.C.

## 1 Introduction

Spontaneous wine fermentation represents a complex ecological process driven by the succession of microbial communities that colonize grape surfaces during ripening. Recent advances in omics-based methodologies have demonstrated that these assemblages are shaped by cultivar, climate, vineyard management and geographical origin, forming what is now widely referred to as the “*microbial terroir*” (Bokulich et al., 2014; Gilbert et al., 2014; Knight et al., 2015; Franco et al., 2024). Vineyard studies further reveal that grape microbiomes are influenced by soil type, canopy microclimate, and agronomic practices (Burns et al., 2016; Mezzasalma et al., 2017), with organic and biodynamic management often enhancing microbial biodiversity (Franco et al., 2024).

In the absence of an inoculated *Saccharomyces cerevisiae* starter, spontaneous fermentation becomes a natural ecological experiment in which oxidative yeasts, filamentous fungi and acetic-acid bacteria dominate the initial phases, before being progressively replaced by fermentative, ethanol-tolerant yeasts (Pinto et al., 2015; Stefanini et al., 2012; Englezos et al., 2022). These early microbial players profoundly influence acidity, volatile aroma composition, phenolic release, and polysaccharide dynamics (Fleet, 2003; Jolly et al., 2014; Mateo and Maicas, 2016; Padilla et al., 2016).

Non-*Saccharomyces* yeasts are increasingly recognized for their contributions to aroma formation, acid modulation, and fermentation robustness (Parlapani et al., 2013; Vilela, 2018; Binati et al., 2020). Species such as *Lachancea thermotolerans*, *Torulaspora delbrueckii*, *Pichia kudriavzevii* and *Schizosaccharomyces pombe* have been shown to contribute to lactic acid formation, ester production, precursor hydrolysis and mannoprotein release (Vejarano, 2020).

Picolit is a traditional sweet wine produced in the Friuli Venezia Giulia region in north-eastern Italy and has been documented since Roman times. The Picolit grape variety is characterized by poor fruit set due to incomplete pollination, resulting in clusters containing only a limited number of berries with a naturally high sugar concentration. In addition, grapes employed for Picolit production are commonly subjected to post-harvest drying either directly on the vine or under controlled environmental conditions, further increasing sugar accumulation. Following harvesting, whole grape clusters or selected berries are gently pressed and the must is cold-decanted prior to fermentation. Alcoholic fermentation may occur spontaneously or be initiated through the addition of selected starter cultures. The wine is subsequently aged in small oak barrels (barriques) for a period ranging from 12 to 24 months (Urso et al., 2008).

In this study, grapes were sourced from Aquila del Torre, an organic and biodynamic winery in Savorgnano del Torre, in the heart of the Friuli Venezia Giulia region, characterized by terraced flysch soils, forest-influenced microclimates and manual vineyard management, conditions known to imprint distinctive microbial biogeographies (Bokulich and Mills, 2013; Stefanini and Cavalieri, 2018). Moreover, biodynamic viticulture has been shown to increase fungal diversity and influence fermentation outcomes (Setati et al., 2015; Capozzi et al., 2015).

Few studies have investigated spontaneous fermentation in Picolit, sweet wine, and none have integrated shotgun metagenomics with metabolomic profiling. This is particularly relevant given the role of *Botrytis cinerea*, whose metabolic activity modulates phenolics, aroma precursors and oxidative pathways (Lopez Pinar et al., 2017; Kelly et al., 2022; Lisov et al., 2023). Understanding microbial succession in this context is essential for clarifying the biological mechanisms underlying the identity of the newly recognized “Friuli Colli Orientali Sottozona Savorgnano D.O.C.”

Here, we present a comprehensive multi-omic characterization of spontaneous Picolit fermentations. Through the integration of shotgun metagenomics, metabolomics and network-based analyses, we describe the dynamics of microbial succession, functional turnover and metabolite restructuring across fermentation, offering new insights into the microbial and chemical factors shaping this distinctive wine.

Given the lack of previous multi-omic investigations on spontaneous Picolit fermentations, the present study was designed as an exploratory ecological characterization aimed at describing microbial and metabolic dynamics occurring during the monitored vinification process rather than establishing causal cultivar-specific effects.

## 2 Materials and methods

### 2.1 Sample collection and fermentation monitoring

Grape must and wine samples were collected by the Azienda Agricola “Aquila del Torre” (Savorgnano del Torre, Povoletto, UD, Italy) during the spontaneous alcoholic fermentation of *Picolit* cultivar at five defined time points (T0, 7/09/24; T1, 9/09/24; T2, 10/09/24; T3, 11/09/24; T5, 14/09/24): prior to fermentation onset (T0) and during fermentation progression (T1, T2, T3, and T5). For each sampling point, three biological replicates were collected, and fermentation progression was monitored by measuring temperature and sugar concentration expressed as°Babo. Sugar levels decreased from > 24°Babo at T0 to approximately 9°Babo at T5, while temperature remained between 19°C and 20°C throughout the monitored fermentation period. Spontaneous fermentation was carried out in 17 hL concrete tanks. Fermentation was conducted without adding SO_2_ and active temperature control. During fermentation, the must was oxygenated in an open vat. No punch-down or pump-over procedures were performed. Fermentation progress was monitored throughout the process according to standard winery practices. Following collection, samples were immediately cooled and transported to the laboratory, where they were processed or stored at −80°C until further analysis.

### 2.2 DNA extraction, quality control and shotgun metagenomic sequencing

Microbial DNA was extracted from must and wine samples using a protocol optimized for complex fermented matrices, ensuring efficient recovery of both bacterial and fungal DNA. Mechanical lysis steps were applied to maximize cell disruption across different microbial taxa using the indication of the according to the manufacturer’s instructions using the DNeasy PowerSoil Pro Kit (Qiagen) afterward centrifugation and washing steps with PBS to take out the possible inhibitors. DNA concentration was quantified using a Qubit fluorometer (Thermo Fisher Scientific), while purity was assessed by spectrophotometric measurements (NanoDrop, Thermo Fisher Scientific). DNA integrity was evaluated by agarose gel electrophoresis. Only samples meeting quality thresholds for concentration, purity, and integrity were retained for sequencing analyses.

High-quality DNA samples were used for shotgun metagenomic sequencing in Nuova Genetica Italiana (NGI) enterprise using the GENEMIND sequencing platform. Libraries were prepared using Illumina-compatible kits following the manufacturer’s instructions and sequenced as paired-end reads (2 × 150 bp). Raw sequencing data were generated in FASTQ format. Sequencing depth was assessed descriptively based on the total number of reads retained after quality filtering and decontamination with Kneaddata, in order to verify suitability for downstream taxonomic and functional analyses

### 2.3 Bioinformatic processing of metagenomic data

#### 2.3.1 Quality control, host decontamination and taxonomy profiling

Raw paired-end reads were initially quality-checked using FastQC (v0.11.9), and quality reports were summarized using MultiQC (v1.14). Pre-processing of sequencing data was performed using Kneaddata (v0.6.1),^1^ an integrated pipeline designed for quality filtering and contaminant removal. Reads mapping against the human reference database (hg37dec_v0.1) and the *Vitis vinifera* reference genome (GCF_000003745.3) were removed using the Bowtie2-based decontamination workflow implemented in Kneaddata. Sequencing depth was evaluated based on the number of reads retained after preprocessing to ensure suitability for downstream taxonomic and functional analyses. No samples were excluded during this step. Taxonomic classification of quality-filtered reads was performed using Kraken2, a k-mer–based classifier that assigns taxonomic labels by exact matching of k-mers to a reference database. Classification was conducted using the PlusPF database. Bracken was applied to re-estimate taxon abundances at species level using the Kraken2 classification reports. The resulting abundance tables were used for all downstream taxonomic analyses.

#### 2.3.2 Functional profiling

Following quality control, metagenomic reads were assembled on a per-sample basis using MEGAHIT, a de Bruijn graph–based assembler optimized for large and complex metagenomic datasets (Li et al., 2015). Assembly was performed using default parameters, generating contigs suitable for gene prediction and functional annotation. Open reading frames (ORFs) were predicted from assembled contigs longer than 500 bp using Prodigal (v2.6.3) (Hyatt et al., 2010). Predicted protein-coding genes from all samples were clustered at 95% sequence identity using CD-HIT (v4.8.1) to generate a non-redundant gene catalog (Fu et al., 2012).

Gene abundance was quantified by mapping quality-filtered paired-end reads from each sample against the non-redundant gene catalog using the KMA alignment tool (Clausen et al., 2018). Read counts were weighted according to target sequence length and subsequently normalized to copies per million (CPM), producing quantitative gene abundance profiles for each sample.

Representative sequences from each gene cluster were functionally annotated using eggNOG-mapper (v2.0.1), which assigns gene functions based on orthology relationships inferred from the eggNOG database. Annotated genes were mapped to multiple functional ontologies, including Gene Ontology (GO), Kyoto Encyclopedia of Genes and Genomes (KEGG) pathways, Clusters of Orthologous Groups (COG), and Pfam/SMART protein domains (The Gene Ontology Resource: 20 years and still GOing strong, 2019).

Custom R scripts were developed to integrate gene abundance data with functional annotations. Separate annotation tables were generated for KEGG pathways and COG categories. KEGG orthology identifiers were mapped to corresponding pathway names using the clusterProfiler package, after which gene abundances were aggregated by pathway by summing CPM values.

For COG annotations, entries containing multiple functional assignments were first expanded into separate rows, followed by aggregation of CPM values by COG category. These processed tables were used for downstream functional profiling and multi-omics integration analyses.

#### 2.3.3 Data filtering and normalization

Metagenomic data were filtered to reduce noise by applying prevalence and abundance criteria. Taxa were retained only if consistently detected across all biological replicates within at least one fermentation time point, while samples with fewer than 1,000 total reads were excluded. Normalization was performed according to downstream analyses. Raw counts were normalized using total sum scaling (TSS) to obtain relative abundances. For diversity analyses and ordination, data were further Hellinger-transformed, while log-transformation was applied when required by specific statistical methods. All filtering and normalization steps were performed in R using base functions and the *vegan* package.

### 2.4 Diversity and community structure analyses

Alpha diversity was assessed using Observed richness, Shannon, and Simpson indices. Differences across fermentation stages were tested using the Kruskal–Wallis test (*p* < 0.05), with Dunn’s *post-hoc* test applied to Observed richness. Beta diversity was evaluated using Bray–Curtis dissimilarities after rarefaction. Community structure was visualized by principal coordinates analysis (PCoA) and non-metric multidimensional scaling (NMDS), and differences among fermentation stages were tested using PERMANOVA. All analyses were performed in R using the *vegan* package.

### 2.5 Differential abundance analysis

Differential abundance analysis was performed to identify microbial taxa exhibiting significant changes across fermentation stages. Pairwise comparisons were conducted between consecutive time points (T0–T1, T1–T2, T2–T3, and T3–T5). To increase robustness and minimize method-specific bias, three complementary approaches were applied: DESeq2, MaAsLin2, and LEfSe.

DESeq2 was applied to raw count data using a negative binomial generalized linear model with fermentation time point as the design factor. Size factors were estimated using the *poscounts* method, and significance was assessed using Wald tests with Benjamini–Hochberg correction (FDR-adjusted *p* < 0.05) (Love et al., 2014).

MaAsLin2 was run using fermentation time point as the sole fixed effect. Features were normalized by total sum scaling and log-transformed prior to modeling. Associations were considered significant at a Benjamini–Hochberg–adjusted *q*-value < 0.05 (Mallick et al., 2021).

LEfSe analysis was performed on species-level relative abundances using default normalization. Differentially enriched taxa were identified using a Kruskal–Wallis test (*p* < 0.05), followed by pairwise Wilcoxon tests (*p* < 0.05), and retained based on a linear discriminant analysis (LDA) score ≥ 2 (Segata et al., 2011).

### 2.6 DART-QDa-driven metabolomic analysis

Must and wine samples collected at each fermentation time point were prepared for metabolomic analysis following a standardized protocol. Samples were processed to remove particulates and analyzed using a Direct Analysis in Real Time (DART) mass spectrometry for comprehensive metabolite detection and quantification. The DART-QDa system consisted of a DART ion source (IonSense, Saugus, MA, United States) coupled to a quadrupole mass spectrometer (Acquity QDa, Waters Corporation, Wimslow, United Kingdom). The QDa detector operated in full scan mode, the cone voltage was set at + 10 V and the sampling frequencies was 5 Hz. Analyses were acquired in positive and negative ionization mode, in the mass range 100-700 m/z. Helium was used as heating gas at a flow rate of 2.5 Lmin-1 and with a temperature of 275°C and 300°C for negative and positive ionization mode, respectively. The MassLynx software version 4.2, provided by Waters Corporation, Wimslow, United Kingdom, was used for the instrument control, data acquisition and data processing. A 5 μL aliquot of sample was directly applied to the Quickstrip sampling device, placed in the moving holder, and exposed to the DART gas stream, enabling the automated analysis of 12 samples within 6 min.

Metabolites were tentatively identified (Sumner et al., 2007) by comparing the corresponding MS spectra with the available literature. Annotated metabolites were compiled into a final data matrix, with metabolites listed in rows and samples in columns. Metabolites were classified into major chemical classes to facilitate biological interpretation, including amino acids and derivatives, sugars and sugar derivatives, phenolic compounds and phenolic acids, flavonoids and anthocyanins, and non-phenolic organic acids.

### 2.7 Multi-omics integration and network analysis

For multi-omics integration, microbial taxa were filtered to retain only features significantly associated with fermentation time and consistently identified as differentially abundant by DESeq2, MaAsLin2, and LEfSe. For each fermentation stage (T0–T5), biological replicates were averaged to obtain a single mean relative abundance per taxon and time point, and metabolomic data were curated accordingly.

Associations between microbial taxa (bacteria and fungi) and metabolites were assessed using Kendall’s tau correlation across fermentation stages. *P*-values were adjusted using the Benjamini–Hochberg false discovery rate (FDR), and associations with FDR-adjusted *p* < 0.1 were retained.

Correlation-based multi-layer networks integrating microbial taxa, metabolites, and metabolic pathways were constructed for each fermentation stage, generating separate networks for bacterial and fungal communities. Networks were built using correlation values as edges and biological features as nodes, with positive and negative correlations represented as distinct edge types.

Module detection was performed using the Louvain community detection algorithm to identify densely connected microbial–metabolic modules. Network construction, visualization, and analysis were carried out in Python using the *networkx*, *matplotlib*, and *plotly* libraries.

### 2.8 Statistical analysis, software, and reproducibility

All statistical analyses were conducted using R (v4.4.1) and Python (v3.12.5). Microbiome analyses were primarily performed using the *phyloseq*, *vegan*, *DESeq2*, *MaAsLin2*, and *microbiomeMarker* packages in R. Functional annotation and pathway analyses were supported by the *clusterProfiler* package. Data manipulation and visualization were carried out using *dplyr*, *tidyr*, *ggplot2*, and related packages.

Network analyses were conducted in Python using *numpy*, *pandas*, *networkx*, *matplotlib*, *plotly*, and the *community* package.

Unless otherwise specified, statistical significance was defined as *p* < 0.05 or FDR-adjusted *p* < 0.05. For the exploratory correlation-based multi-omics integration analyses, a more permissive threshold (FDR-adjusted *p* < 0.1) was applied to retain potentially biologically relevant associations for hypothesis generation and network visualization. These associations were interpreted cautiously and not considered evidence of direct ecological interactions.

The entire computational workflow was designed to be fully reproducible. All scripts were organized into modular components and executed through a master script that orchestrates the complete analysis pipeline. Input data, intermediate files, and outputs were systematically managed to ensure traceability and reproducibility of results.

## 3 Results

### 3.1 Fermentation progression and chemical characterization

The chemical characterization of samples collected during spontaneous fermentation revealed a progressive consumption of fermentable sugars over time. Sucrose, glucose, and fructose all showed a marked decrease from T0 to T5, indicating the advancement of alcoholic fermentation (Table 1). In parallel, the summed sugar fractions also declined substantially, further supporting the progressive depletion of the carbohydrate pool. Acetic acid remained low throughout the process, although a slight increase was observed at the last fermentation stages collected. These chemical changes are consistent with the microbial and metabolomic shifts observed across fermentation.

**TABLE 1:** Chemical characterization of Picolit must and wine samples collected at different stages of spontaneous fermentation.

Time point	Glucose	Fructose	Sucrose	Acetic acid
**T0**	70.17	113.30	46.15	0.02
**T1**	64.90	112.73	43.86	0.10
**T2**	64.07	109.80	42.47	0.07
**T3**	43.63	89.23	31.43	0.11
**T5**	17.68	16.15	10.50	0.14

Concentrations of sucrose, glucose (D-Glu), fructose, and acetic acid were measured at each sampling time point (T0, T1, T2, T3, and T5) during spontaneous fermentation. Values are expressed as g/L.

### 3.2 Dataset overview and sequencing quality

Shotgun metagenomic sequencing generated a total of 7.5 × 10^8^ raw reads across the 15 samples analyzed, with sequencing depth ranging from 436,292 to 57,044,757 reads per sample (mean = 25.0 million reads, median = 26.7 million reads). Following quality filtering and host-read decontamination with Kneaddata, the number of cleaned reads ranged from 315,028 to 42,298,782 per sample, with a mean of 19.7 million reads (SD = 13.4 million) and a median of 20.1 million reads per sample. Quality control analyses indicated that preprocessing substantially improved per-base sequence quality and GC-content profiles, ensuring suitability for downstream taxonomic and functional analyses. A total of 1,469 microbial taxa were detected in the unfiltered dataset. After removing low-abundance and low-prevalence taxa, 932 taxa were retained. The original abundance distribution was right-skewed, dominated by low-prevalence taxa, whereas prevalence analysis showed a small core microbiota accompanied by a long tail of rare, sample-specific taxa (Supplementary Figure 1).

### 3.3 Microbial diversity across fermentation

**FIGURE 1:**
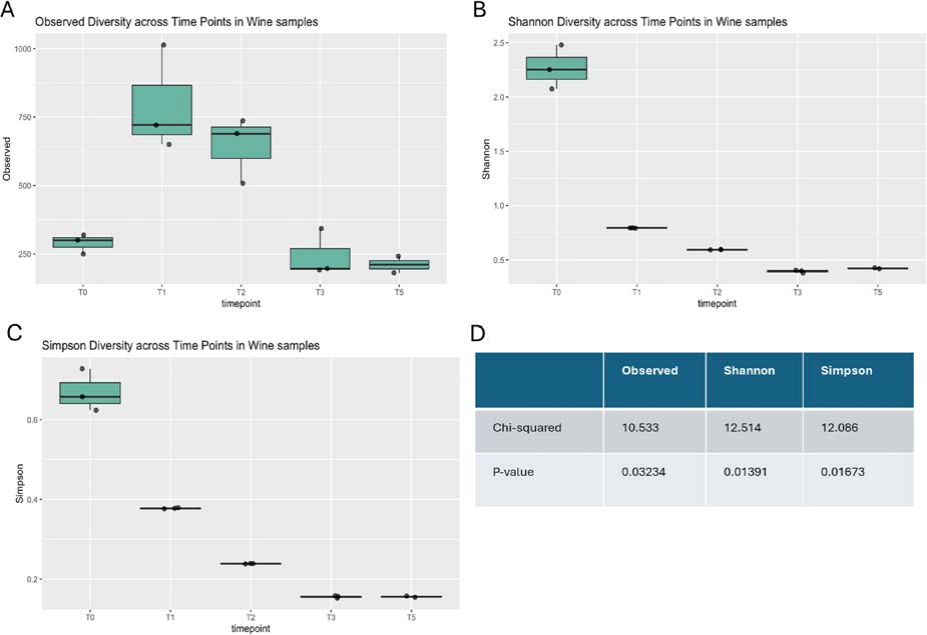
Alpha diversity indices of samples during fermentation and results of the Kruskal–Wallis test. **(A)** Boxplot of Observed richness, representing the number of unique taxa detected in each sample across fermentation stages. **(B)** Boxplot of Shannon diversity, summarizing richness and evenness for each sample. **(C)** Boxplot of Simpson diversity, representing evenness-weighted diversity across samples. **(D)** The table reports the Kruskal–Wallis test statistics and associated *p*-values for the three indices.

Observed richness transiently increased from T0 to T1 before progressively declining throughout fermentation. This pattern may reflect early ecological succession dynamics occurring at the onset of spontaneous fermentation, although technical factors such as sequencing depth and detection sensitivity may also have contributed to the observed variation. Differences in alpha-diversity indices across fermentation stages were assessed using the Kruskal–Wallis test, revealing significant differences among time points (*p* < 0.05) (Figure 1).

**FIGURE 2:**
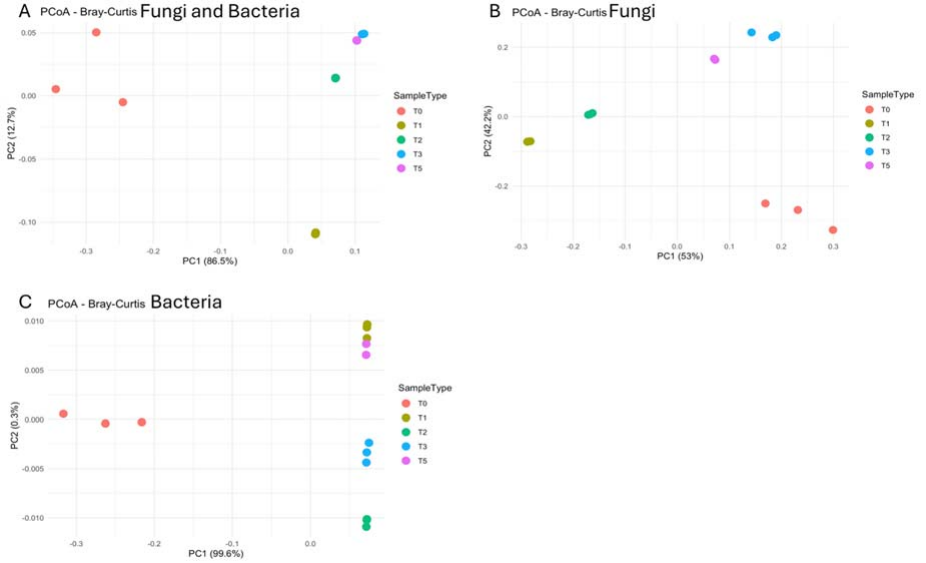
Principal Coordinates Analysis (PCoA) of microbial communities across fermentation stages. **(A)** the PCoA based on Bray–Curtis dissimilarities including both fungal and bacterial taxa. Samples cluster clearly according to fermentation stage, with early stages (T0–T1) separated from mid- and late-fermentation communities (T2–T5). PC1 (86.5%) and PC2 (12.7%). **(B)** The PCoA restricted to *fungal taxa* at different fermentation stages. The first two axes explain 53% and 42.2% of variance, **(C)** The PCoA of *bacterial taxa*, where community structure is primarily driven by a single axis (PC1 = 99.6%).

PCoA based on Bray–Curtis dissimilarities showed clear temporal clustering of samples (Figure 2A). Early stages (T0–T1) were distinct from mid and late monitored fermentation (T2–T5), with PC1 explaining 86.5% of overall variance. Fungal communities exhibited moderate successional structuring (Figure 2B), whereas bacterial communities collapsed into a single dominant axis (PC1 = 99.6%; Figure 2C).

NMDS ordinations confirmed stage-specific microbial assemblages for fungi (Supplementary Figures 2), with early samples exhibiting greater dispersion and later samples forming compact clusters. Fermentation stage explained > 98% of variance in community structure for mixed, fungal-only, and bacterial-only datasets (*p* = 0.001).

### 3.4 Taxonomic composition and temporal dynamics

**FIGURE 3:**
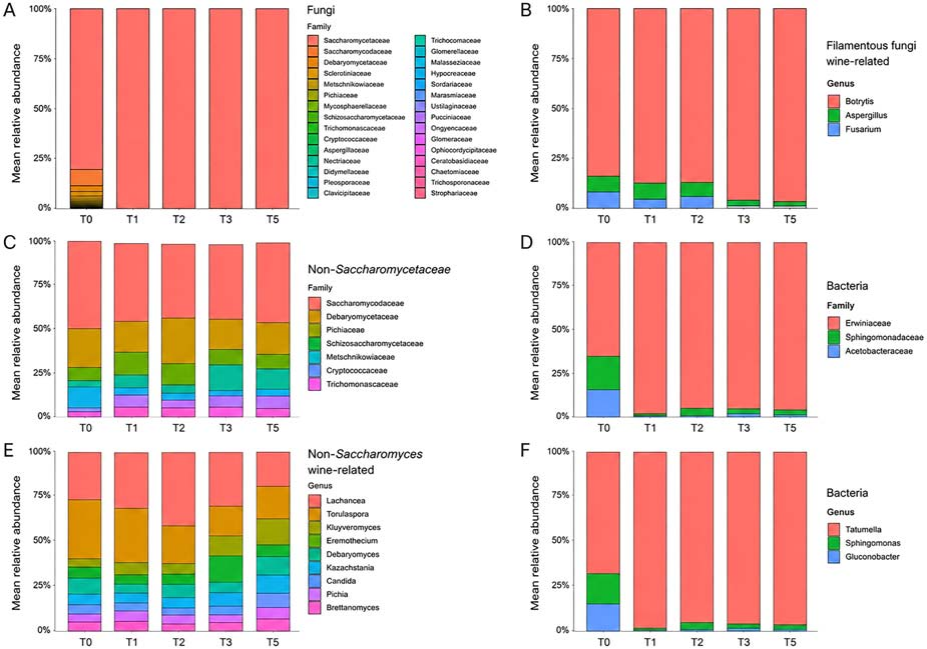
Taxonomic composition of fungal and bacterial communities across fermentation stages. Relative abundance profiles of fungal and bacterial communities across fermentation stages. Stacked bar plots showing the mean relative abundance of microbial taxa detected during spontaneous fermentation at different taxonomic levels. Panels report the composition of the **(A)** overall fungal community at family level, **(B)** filamentous wine-related fungi at genus level, **(C)** non-Saccharomycetaceae families, **(D)** bacterial families, **(E)** wine-related non-*Saccharomyces* genera, and **(F)** dominant bacterial genera across fermentation stages (T0–T5).

Across all stages, as is showed in Figure 3 the fungal community was dominated by members of the Saccharomycetaceae. In the early phases of fermentation (T0–T1), however, a broader diversity of non-*Saccharomyces* families was detected, including Saccharomycodaceae, Metschnikowiaceae, Debaryomycetaceae and Pichiaceae. Several wine-associated genera, *Lachancea*, *Torulaspora*, *Pichia*, and *Debaryomyces*, were present at relative abundance over the 0.5% from T0 to T2. Additional, less common fungal genera such as *Komagataella*, *Kwoniella*, *Australozyma*, and *Lodderomyces* were also observed with variable relative abundance across samples. Filamentous fungi were largely represented by *Botrytis cinerea* (Sclerotiniaceae), with minor contributions from *Aspergillus* and *Fusarium*.

Regarding the bacterial community, early fermentation (T0–T1) was dominated by *Tatumella* (Erwiniaceae), accompanied by taxa belonging to Sphingomonadaceae and Acetobacteraceae (Figure 3). Most bacterial groups exhibited a marked decline after T1, consistent with the progression of fermentation.

### 3.5. Abundance trajectories of dominant taxa

**FIGURE 4:**
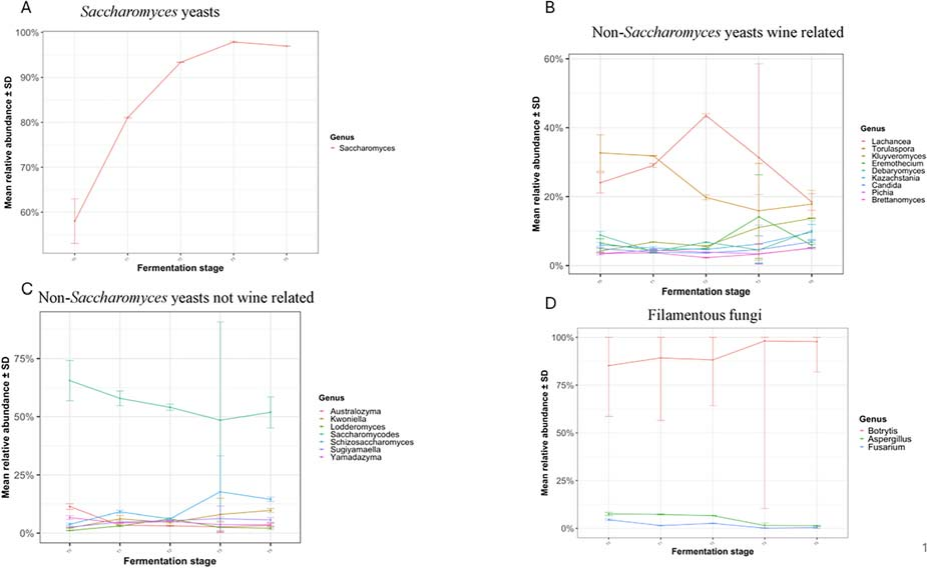
Temporal trajectories of *Saccharomyces cerevisiae*, non-*Saccharomyces* yeasts, and wine-related filamentous fungi across fermentation stages. Line plots showing **(A)** the mean relative abundance of *Saccharomyces cerevisiae* across fermentation stages, **(B)** the temporal trends of the main non-*Saccharomyces* yeast genera, **(C)** the temporal trends of the main non-*Saccharomyces* yeast genera not related with wine and **(D)** the trajectories of wine-related molds and filamentous fungi, including *Botrytis*, *Aspergillus*, and *Fusarium*.

The relative abundance of *Saccharomyces* spp. increased steadily from T1 onward and reached its highest levels between T3 and T5. Several non-*Saccharomyces* genera, particularly *Debaryomyces*, *Pichia*, and *Schizosaccharomyces*, persisted beyond the early stages and displayed distinct abundance peaks between T1 and T3. Among filamentous fungi, *Botrytis cinerea* showed a continuous increase across fermentation, whereas other molds declined sharply after the earliest time points. Bacterial trajectories were characterized by the dominance and persistence of *Tatumella*, while oxidative genera such as *Gluconobacter* and *Sphingomonas* were no longer detected after T1 (Figure 4).

### 3.6 Differential abundance analyses

**FIGURE 5:**
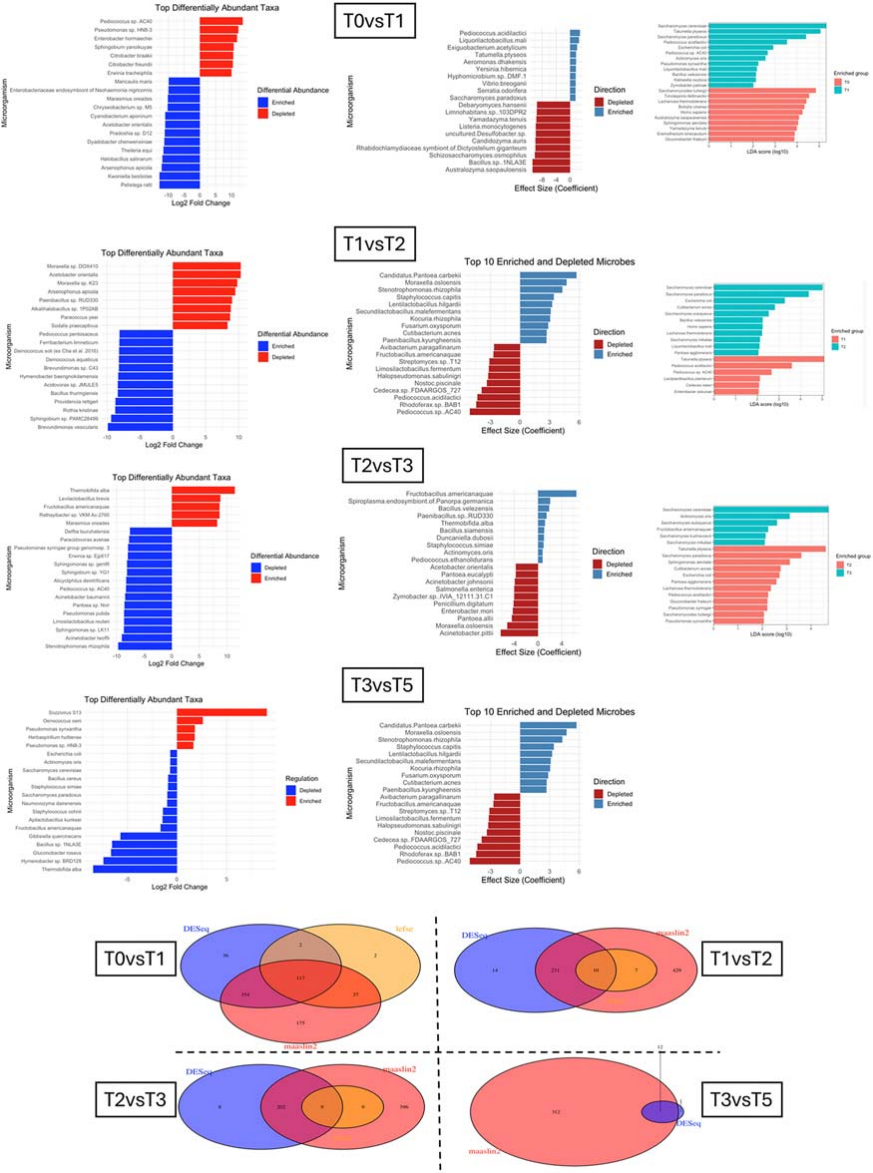
Differential abundance analyses across fermentation stages using DESeq2, MaAsLin2, and LefSe. The figure reports the results of differential abundance analyses performed between consecutive fermentation stages (T0–T1, T1–T2, T2–T3, T3–T5) using DESeq2, MaAsLin2, and LEfSe. For each comparison, the plots display the taxa identified as significantly enriched at either stage according to the statistical criteria of the three methods. Venn diagrams summarize the overlap among DESeq2, MaAsLin2, and LEfSe, indicating the number of taxa consistently detected as differentially abundant across tools.

Differential abundance analysis performed with DESeq2, MaAsLin2, and LEfSe identified a set of taxa exhibiting significant stage-specific enrichment across fermentation (Figure 5). The highest number of enriched taxa was detected at T0, including several *Gluconobacter* species, multiple non-*Saccharomyces* yeasts, and *Botrytis cinerea* (Table 2). During mid-fermentation (T1–T3), the enriched taxa were predominantly *Saccharomyces* species (Table 3). No fungal taxa showed significant enrichment at the last stage collected at the end of the fermentation process (T5).

**TABLE 2:** List of differentially abundant microorganisms, time T0-T2.

	T0 vs. T1		T1 vs. T2	
Enriched	T0	T1	T1	T2
**Bacteria**	*Gluconobacter (frateurii, cerinus, thailandicus, oxydans, sphaericus, albidus, roseus)*	*Tatumella ptyseos*	*Tatumella ptyseos*	
***Saccharomyces* yeasts**	*Saccharomyces kudriavzevii*	*Saccharomyces cerevisiae* *Saccharomyces paradoxus*		*Saccharomyces eubayanus* *Lachancea thermotolerans*
**Non-*Saccharomyces* yeasts**	*Lachancea thermotolerans, Saccharomycodes ludwigii, Torulaspora delbrueckii, Torulaspora globosa, Pichia kudriavzevii, Debaryomyces hansenii, Schizosaccharomyces pombe* *Schizosaccharomyces osmophilus* *Australozyma saopauloensis* *Sugiyamaella lignohabitans* *Komagataella phaffii* *Kwoniella (dendrophila, bestiolae, botswanensis, newhampshirensis)*			
**Molds/filamentous fungi**	*Botrytis cinerea* *Cercospora beticola*			

**TABLE 3:** List of Differentially Abundant Microorganisms, time T2–T5.

	T2 vs. T3		T3 vs. T5	
Enriched	T2	T3	T3	T5
**Bacteria**	*Sphingomonas aerolata*		*Gluconobacter roseus*	
**Saccharomyces yeasts**	*Saccharomyces paradoxus*	*Saccharomyces cerevisiae* *Saccharomyces eubayanus* *Saccharomyces kudriavzevii* *Saccharomyces mikatae*		

#### 3.7 Functional potential and pathway dynamics

**FIGURE 6:**
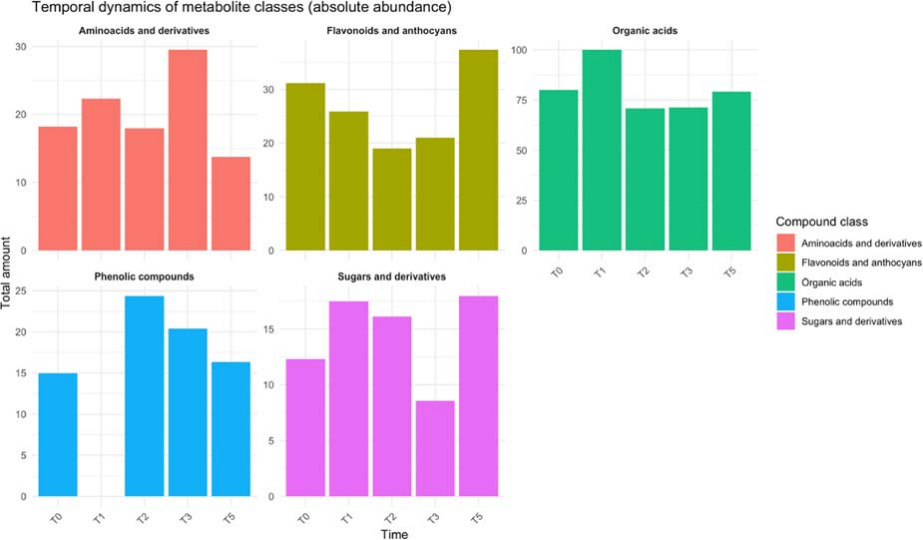
Temporal dynamics of major metabolite classes across fermentation stages. Stacked bar plots showing the summed absolute abundances of metabolites grouped into major compound classes (amino acids and derivatives, flavonoids and anthocyanins, phenolic compounds, sugars and derivatives, organic acids) across fermentation stages (T0, T1, T2, T3, T5). Each bar represents the total abundance of all metabolites assigned to a given class at each time point.

The temporal dynamics of major metabolite classes (Figure 6), together with the metabolite-level trends within each class (Supplementary Figure 6), revealed structured, stage-dependent shifts in the chemical composition of the must throughout fermentation. Organic acids represented the most abundant class and displayed a biphasic trend, with a pronounced increase at T1 followed by a reduction during mid-fermentation and a subsequent rise at T5, reflecting the combined effects of microbial metabolism and chemical transformations linked to sugar consumption and acid turnover. Sugars and sugar derivatives showed the expected decline as fermentation progressed, with a sharp decrease between T2 and T3 corresponding to the period of highest fermentative activity. Phenolic compounds were undetectable at T1 but reappeared from T2 onward, suggesting early-phase degradation or transformation followed by reaccumulation or release from glycosylated forms. Flavonoids and anthocyanins decreased during initial stages and exhibited a marked increase at T5, consistent with late-stage release or stabilization processes. Amino acids and their derivatives showed moderate fluctuations, with the highest cumulative abundance observed at T3, indicating active nitrogen turnover and yeast-associated metabolic processing. Overall, the combined metabolite trajectories illustrate the progressive restructuring of the chemical landscape as fermentation advances and reflect the metabolic activities of dynamically evolving microbial communities throughout the fermentation process (Supplementary Figure 6).

The most pronounced restructuring concerning the functional profiling based on EggNOG and KEGG annotations occurred between T0 and T1, where 20 pathways were significantly enriched in T1, while 9 remained characteristic of the initial must at T0 (Supplementary Figure 3A and Table 4). This transition marks the phase with the highest functional turnover within the dataset.

**TABLE 4:** Number of enriched pathways for each time point.

Comparison	Enriched in Tx	N pathways
**T0 vs. T1**	T0	9
**T0 vs. T1**	T1	20
**T1 vs. T2**	T1	5
**T1 vs. T2**	T2	6
**T2 vs. T3**	–	0
**T3 vs. T5**	T3	9
**T3 vs. T5**	T5	8

Changes were also detected in several COG functional categories (Supplementary Figure S3B and Table 5). Early fermentation (T0–T1) was characterized by higher representation of pathways associated with the degradation of aromatic and xenobiotic compounds. In contrast, mid- to late-fermentation stages (T2–T5) showed an increased abundance of pathways related to cellular stress responses, including osmotic and oxidative stress functions.

**TABLE 5:** Number of Enriched COG categories for each time point.

Comparison	Enriched in Tx	N COG categories
**T0 vs. T1**	T0	9
**T0 vs. T1**	T1	7
**T1 vs. T2**	T1	5
**T1 vs. T2**	T2	4
**T2 vs. T3**	–	0
**T2 vs. T5**	T2	8
**T2 vs. T5**	T5	1
**T3 vs. T5**	–	0

Pairwise comparisons of selected functional groups, aroma-related pathways, oxidative-stress pathways, and biofilm-associated pathways, highlighted clear temporal patterns (Supplementary Figures 4A–C). Between T0 and T1, pathways involved in aromatic-compound degradation markedly decreased, while pathways related to stress adaptation and biofilm formation became more represented. Between T1 and T2, a subset of aromatic-degradation pathways persisted, although at lower overall abundance. Finally, the comparison between T3 and T5 revealed stabilization of oxidative-stress pathways and a further decline of bacterial biofilm-associated pathways, consistent with the advanced stage of fermentation.

### 3.8 Integration of microbial–metabolite–pathway networks

Correlation-based network analysis revealed distinct stage-specific associations linking microbial taxa, metabolites, and functional pathways (Supplementary Figures 5A,B, 7A,B). The network at T0 displayed the highest degree of complexity, characterized by numerous interconnected nodes and multiple co-occurring microbial and metabolic features. In contrast, networks at T1 and T3 showed a marked reduction in the number of connections, resulting in fewer, more compact and clearly defined functional modules. This progressive simplification reflects the restructuring of microbial associations across fermentation stages.

## 4 Discussion

The spontaneous fermentation of Picolit described in this study reveals a rapidly evolving microbial ecosystem shaped by ecological selection pressures and potentially associated with the physicochemical characteristics of the fermenting must. Although T0 represents the initial expression of the vineyard-derived epiphytic microbiota, the Observed richness increases at T1. This trend likely reflects not only the activation or detection of additional taxa at the onset of fermentation, but also the reduced between-replicate variability observed at T1. As shown by beta-diversity analyses, the high dispersion among T0 replicates results in many low-prevalence or inconsistently detected taxa being removed during preprocessing, thereby lowering apparent richness at this stage. At T1, the microbiota begins to follow a more defined ecological trajectory, taxa become more consistently shared across replicates, and fewer features fall below filtering thresholds. This transient increase in detectable diversity is commonly observed in spontaneous fermentations before ecological filtering reduces community complexity at later stages as expected (Bokulich et al., 2014; Gilbert et al., 2014; Knight et al., 2015; Belda et al., 2017). Similar observations from vineyard-scale microbiome studies further demonstrate how soil, canopy microclimate, agronomic practices and landscape structure shape the initial microbial inoculum entering spontaneous fermentations (Burns et al., 2016; Mezzasalma et al., 2017). The significant reduction in diversity throughout fermentation, confirmed by alpha-diversity statistics, aligns with the progressive filtering effect typical of spontaneous fermentations (Pinto et al., 2015; Wang et al., 2015), in which rising ethanol, nutrient depletion and oxygen limitation favor a restricted subset of fermentative yeasts. The broad fungal and bacterial taxa detected at T0, including numerous non-*Saccharomyces* yeasts, filamentous fungi and oxidative bacteria, reflect what Franco et al. (2024) describe as the “*microbial terroir*,” a complex ensemble of microorganisms shaped by vineyard soil, climate, and canopy management (Zarraonaindia et al., 2015; Morgan et al., 2017; Franco et al., 2024).

The persistence of *Botrytis cinerea* observed during Picolit fermentation may be associated with the high-sugar environment characteristic of this cultivar, although comparative studies will be required to confirm cultivar-specific effects. In many spontaneous fermentations, *B. cinerea* declines rapidly throughout the monitored vinification period, yet here it maintains a dominant role, likely due to the natural acinellatura of Picolit, which results in fewer berries per cluster and elevated sugar concentration (Zulini et al., 2005). This microenvironment not only supports *B. cinerea* persitance but also modulates its metabolic activity under osmotic stress conditions (Zakrzewska et al., 2011). The influence of *B. cinerea* on must chemistry is well documented: it produces oxidative enzymes capable of degrading phenolics and aromatic compounds (Lopez Pinar et al., 2017), and can significantly alter both aroma composition and sensory quality (Marchal et al., 2020; Lisov et al., 2023). Its persistence may indicate prolonged ecological relevance during fermentation within the fermentative ecosystem with both beneficial and detrimental enological consequences. Beneficial effects include the promotion of glycosidic hydrolysis and the liberation of aromatic precursors typical of noble rot wines (Kelly et al., 2022), potentially contributing to the honeyed, floral and dried-fruit notes described in Picolit. However, its oxidative activity can reduce phenolic stability and increase susceptibility to browning. The sustained abundance of *B. cinerea* observed here therefore suggests a fermentation system in which Botrytis-driven metabolic processes extend beyond the typical pre-fermentative phase.

The early stages of fermentation are further characterized by a rich and functionally diverse assemblage of non-*Saccharomyces* yeasts. This group plays a central role in shaping the initial chemical transformations of the must, in line with previous observations that highlight their importance in spontaneous fermentations (Jolly et al., 2014; Mateo and Maicas, 2016; Padilla et al., 2016). Genera such as *Lachancea*, *Pichia*, *Torulaspora*, *Debaryomyces*, and *Schizosaccharomyces* were abundant in T0–T2 and displayed stage-specific trajectories indicative of active metabolic participation. *Lachancea thermotolerans*, which peaked between T0 and T1, is known to convert sugars into lactic acid and lower pH (Vilela, 2018; Englezos et al., 2022). This feature is particularly relevant for Picolit, where the naturally high sugar content associated with acinellatura produces must that benefit from early acidification to maintain balance and freshness. *Pichia kudriavzevii*, enriched at T0-T1, is recognized for its pectinolytic and glycosidase activities (González et al., 2006; Binati et al., 2020), which can facilitate the release of aroma precursors and influence mouthfeel. Species of *Schizosaccharomyces* show an uncommon persistence into mid and late monitored fermentation, consistent with their ethanol tolerance, malic acid metabolism, and ability to ferment alternative substrates (Jolly et al., 2014; Benito, 2019; Englezos et al., 2022). Less commonly described genera such as *Komagataella* and *Kwoniella* appear to play roles as well: their negative correlations with several flavonoids and phenolic acids suggest involvement in early phenolic depletion, consistent with their oxidative or degradative potential described in other spontaneous fermentations (Giang and Khai, 2023). These observations echo the wide metabolic repertoire of rare non-*Saccharomyces* species highlighted by Bokulich et al. (2016) and reinforce the importance of taxa often overlooked in enological studies (Bokulich et al., 2016).

The transition to fermentation dominated by *Saccharomyces* species between T2 and T3 represents the turning point of the microbial succession. The rise of *S. cerevisiae*, together with *S. paradoxus*, *S. eubayanus*, *S. kudriavzevii*, and *S. mikatae*, suggests a multi-species fermentative consortium, a phenomenon increasingly recognized in spontaneous fermentations (Ludlow et al., 2016; Belda et al., 2017). In parallel, the bacterial community collapses almost entirely after T1, as ethanol and anaerobiosis eliminate most oxidative and epiphytic bacteria (Osborne and Edwards, 2005). This ecological reshaping corresponds to a profound shift in functional potential: pathways associated with aromatic and xenobiotic degradation, typical of the epiphytic phase, are replaced by stress-response systems, sugar transport machinery and pathways linked to survival under ethanol accumulation (Hacquard et al., 2015). Importantly, the monitored fermentation process reflects the production style of Picolit as a naturally sweet wine, in which fermentation is intentionally interrupted before complete sugar depletion to preserve residual sweetness and sensory balance. Consequently, the late fermentation stages analyzed in this study should not be interpreted as the endpoint of a fully completed dry alcoholic fermentation, but rather as the advanced stages of a controlled sweet-wine vinification process characterized by persistent high sugar concentrations and prolonged osmotic stress conditions. These conditions likely contribute to the unusual persistence of specific microbial taxa and metabolic activities observed throughout fermentation. The metabolomic data mirror these transitions: the disappearance of phenolics at T1 and their reappearance at T2–T3 reflect the combined activities of degradative microbes present early on (e.g., *Gluconobacter*, *Kwoniella*) and the stabilizing influence of fermentative yeasts later in the process.

Exploratory multi-omic network analysis (Supplementary Figure 7) suggested coordinated shifts in microbial, metabolic, and functional associations across fermentation stages. The network analyses were retained primarily as a visual integrative framework supporting the temporal restructuring observed independently through diversity, taxonomic, and metabolomic analyses. At T0, the network is highly modular, comprising numerous interconnected microorganisms and metabolites, indicative of an open and heterogeneous ecosystem. At T1, the network collapses into a smaller structure centered on *Saccharomyces paradoxus* and stress-related pathways. By T3, *Saccharomyces kudriavzevii* emerges as a central node in a compact, functionally specialized network dominated by oxidative phosphorylation, aromatic degradation and amino acid–related pathways. This reduction in inferred network complexity was consistent with the progressive reduction in microbial diversity observed during fermentation.

Overall, the spontaneous fermentation of Picolit emerges as a distinct and biologically rich system shaped by three key forces: the high initial biodiversity of the vineyard-derived microbiota, the unusual and persistent presence of *Botrytis cinerea*, and the coordinated activities of non-*Saccharomyces* and *Saccharomyces* yeasts during fermentation. These observations suggest that spontaneous fermentation, together with the absence of SO_2_ addition, may contribute substantially to the chemical complexity and sensory identity associated with Picolit wines. Despite the breadth of the multi-omic dataset and the integration of metagenomic, taxonomic and metabolomic approaches, several limitations must be acknowledged. The observational nature of shotgun metagenomics restricts the ability to draw causal inferences: correlations between microorganisms, metabolites and pathways suggest potential biological interactions but do not confirm them. Similar limitations have been noted in other wine microbiome studies (Bokulich et al., 2016; Belda et al., 2017), highlighting the need for controlled microfermentations, strain isolation and functional assays to validate the hypotheses generated here. Functional predictions derived from KEGG and EggNOG annotations indicate metabolic potential but cannot confirm gene expression; transcriptomic or metatranscriptomic analyses would be required to determine which pathways were actively expressed at each fermentation stage. The untargeted metabolomics workflow used, while comprehensive, is less sensitive to volatile and highly reactive aroma compounds, meaning that certain fermentation-derived metabolites, particularly terpenes, norisoprenoids and thiols, may have been underrepresented. Moreover, because DART-MS analyses are performed without chromatographic separation and are sensitive to matrix-dependent ionization effects, the intermittent detection of low-abundance metabolites should be interpreted cautiously. Therefore, compounds reported as ‘not detected’ may reflect concentrations below the analytical detection threshold under the applied conditions rather than absolute absence. A further limitation concerns the extremely high microbial richness observed at T0–T1: while filtering low-prevalence taxa is necessary for robust statistical analysis, it may inadvertently remove rare taxa with non-negligible metabolic roles. An additional limitation of the present study is the absence of parallel control fermentations from other grape cultivars or wineries from the same vintage, which prevents definitive attribution of the observed microbial dynamics specifically to Picolit physiology or acinellatura-associated sugar levels.

## 5 Conclusion

This study provides a comprehensive multi-omic characterization of spontaneous Picolit fermentation, highlighting a structured ecological succession in which an initially diverse vineyard-derived microbiota progressively shifts toward a fermentation-adapted community. The persistence of *Botrytis cinerea* throughout the monitored vinification period, together with the early activity of several non-*Saccharomyces* yeasts, was associated with marked changes in metabolite composition, including phenolic-associated features and pathways linked to aroma precursor transformation. As fermentation progressed, multiple *Saccharomyces* species became dominant, coinciding with a progressive restructuring of the functional and metabolic landscape. The integration of metagenomic and metabolomic data revealed dynamic microbial and functional changes occurring during spontaneous Picolit vinification together with the absence of SO_2_ addition supports the hypothesis that grape physiology, native microbiota, and fermentation environment collectively contribute to shaping the observed fermentation trajectory. Further comparative studies including additional grape cultivars, wineries, and vintages will be necessary to determine the extent to which these microbial and metabolic patterns are specific to Picolit and its acinellatura-associated high-sugar environment.

## Data Availability

Raw sequencing data have been deposited in the European Nucleotide Archive (ENA) under the accession number PRJEB110419 and are publicly available at: https://www.ebi.ac.uk/ena/browser/view/PRJEB110419. In order to ensure reproducibility and facilitate reuse, the scripts supporting the analyses presented in this work have been deposited in a publicly accessible GitHub repository: https://github.com/micheladellalma/MultiOmicsFermentation.

## Footnotes

1
https://bitbucket.org/biobakery/kneaddata

## Supplementary material

Supplementary Figure S1 Histogram of distribution of taxon. **(A)** Abundance-based filtering threshold for taxa inclusion. Histogram displaying the log10-transformed abundance distribution of all taxa prior to filtering. The vertical dashed blue line denotes the minimum abundance threshold applied to remove extremely low-count taxa that may derive from sequencing noise or sporadic detection. Taxa to the left of the threshold are excluded from downstream analyses, whereas those to the right are retained. **(B)** Prevalence-based filtering threshold for taxa inclusion. Histogram showing how many samples contain each taxon. The dashed blue line marks the prevalence cutoff. Taxa detected in fewer samples than this threshold are removed to ensure statistical robustness and minimize false positives driven by ultra-rare features. The retained taxa represent a more reliable subset of the microbial community.

Supplementary Figure S2 NMDS ordination of fungal and bacterial communities during spontaneous fermentation. NMDS ordination based on Bray–Curtis dissimilarities of the fungal community detected across fermentation stages. Labels “sp.n” indicate individual species ordination identifiers, where n corresponds to the species number. Fermentation stages are indicated by numbers 0–5, corresponding to the sampled fermentation time points (T0–T5).

Supplementary Figure S3 Differential pathway (KEGG) and functional (COG) analyses across fermentation stages. **(A)** A bubble plot of KEGG pathways identified as differentially abundant between consecutive fermentation stages; each point represents a pathway, with color indicating the stage in which it is enriched and bubble size reflecting the corresponding LDA effect size. **(B)** A bubble plot of differentially abundant COG functional categories derived from MaAsLin2 models; each point represents a COG category, positioned along the x-axis according to the model coefficient, with bubble size indicating statistical significance. Only pathways and categories meeting the predefined significance thresholds are reported.

Supplementary Figure S4 Pairwise comparisons of pathway-level functional potential across fermentation stages. The figure reports KEGG pathway abundances for each pairwise comparison between fermentation stages (T0–T1, T1–T2, T3–T5). Bar plots display the log10-transformed read counts associated with each pathway, grouped by functional category (aromatic metabolism, oxidative stress, biofilm formation). Error bars indicate variation among replicates. Pathways enriched in the earlier or later stage of each comparison are shown according to the direction of differential abundance.

Supplementary Figure S5 Correlation networks between microbial taxa and metabolic pathways. Network graphs illustrating significant Spearman correlations between microbial taxa and KEGG pathways across fermentation stages. **(A)** The figure shows correlations for fungal taxa; nodes represent fungal genera or species and metabolic pathways, with edges indicating significant positive or negative associations. **(B)** The figure displays the corresponding bacterial correlation network, using the same node and edge conventions. Only correlations meeting the predefined statistical thresholds are included.

Supplementary Figure S6 Temporal variation of individual metabolite groups across fermentation stages. Line plots and stacked bar plots illustrating the temporal patterns of major metabolite groups across fermentation stages. Each panel reports, for a specific compound class, the relative abundances (stacked bar plots) and absolute abundances (line plots) of individual metabolites: amino acids and derivatives; sugars and their derivatives; phenolic compounds; flavonoids and anthocyanins; non-phenolic organic acids. Each plot depicts metabolite-level trajectories from T0 to T5, based on summed or individual abundance values within each compound class.

Supplementary Figure S7 Multi-layer microbial–metabolite–pathway networks across fermentation stages. Network diagrams showing significant associations among microbial taxa, metabolites, and KEGG pathways at different fermentation stages. Panel displays the main network modules identified at T0, T1, T2, and T3, highlighting the stage-specific organization of microbial, metabolic, and functional interactions. With line red positive correlation, in blue the negative correlation.

## References

[B1] Belda I. Zarraonaindia I. Perisin M. Palacios A. Acedo A. (2017). From vineyard soil to wine fermentation: microbiome approximations to explain the “terroir”, Concept. *Front. Microbiol.* 8:821. 10.3389/fmicb.2017.00821 28533770 PMC5420814

[B2] Benito S. (2019). The management of compounds that influence human health in modern winemaking from an HACCP point of view. *Fermentation* 5:33. 10.3390/fermentation5020033

[B3] Binati R. L. Lemos Junior W. J. F. Luzzini G. Slaghenaufi D. Ugliano M. Torriani S. (2020). Contribution of non-Saccharomyces yeasts to wine volatile and sensory diversity: a study on *Lachancea thermotolerans*, Metschnikowia spp. and *Starmerella bacillaris* strains isolated in Italy. *Int. J. Food Microbiol.* 318:108470. 10.1016/j.ijfoodmicro.2019.108470 31841784

[B4] Bokulich N. A. Collins T. S. Masarweh C. Allen G. Heymann H. Ebeler S. E. et al. (2016). Associations among wine grape microbiome, metabolome, and fermentation behavior suggest microbial contribution to regional wine characteristics. *mBio* 7:e00631-16. 10.1128/mBio.00631-16 27302757 PMC4959672

[B5] Bokulich N. A. Mills D. A. (2013). Improved selection of internal transcribed spacer-specific primers enables quantitative, ultra-high-throughput profiling of fungal communities. *Appl. Environ. Microbiol.* 79 2519–2526. 10.1128/AEM.03870-12 23377949 PMC3623200

[B6] Bokulich N. A. Thorngate J. H. Richardson P. M. Mills D. A. (2014). Microbial biogeography of wine grapes is conditioned by cultivar, vintage, and climate. *Proc. Natl. Acad. Sci. U. S. A.* 111 E139–E148. 10.1073/pnas.1317377110 24277822 PMC3890796

[B7] Burns K. N. Bokulich N. A. Cantu D. Greenhut R. F. Kluepfel D. A. O’Geen A. T. et al. (2016). Vineyard soil bacterial diversity and composition revealed by 16S rRNA genes: differentiation by vineyard management. *Soil Biol. Biochem.* 103 337–348. 10.1016/j.soilbio.2016.09.007

[B8] Capozzi V. Garofalo C. Chiriatti M. A. Grieco F. Spano G. (2015). Microbial terroir and food innovation: the case of yeast biodiversity in wine. *Microbiol. Res.* 181 75–83. 10.1016/j.micres.2015.10.005 26521127

[B9] Clausen P. T. L. C. Aarestrup F. M. Lund O. (2018). Rapid and precise alignment of raw reads against redundant databases with KMA. *BMC Bioinformat.* 19:307. 10.1186/s12859-018-2336-6 30157759 PMC6116485

[B10] Englezos V. Jolly N. P. Di Gianvito P. Rantsiou K. Cocolin L. (2022). Microbial interactions in winemaking: ecological aspects and effect on wine quality. *Trends Food Sci. Technol.* 127 99–113. 10.1016/j.tifs.2022.06.015

[B11] Fleet G. (2003). Yeast interactions and wine flavour. *Int. J. Food Microbiol.* 86 11–22. 10.1016/S0168-1605(03)00245-9 12892919

[B12] Franco G. C. Leiva J. Nand S. Lee D. M. Hajkowski M. Dick K. et al. (2024). Soil microbial communities and wine terroir: research gaps and data needs. *Foods* 13:2475. 10.3390/foods13162475 39200402 PMC11354026

[B13] Fu L. Niu B. Zhu Z. Wu S. Li W. (2012). CD-HIT: accelerated for clustering the next-generation sequencing data. *Bioinformatics* 28 3150–3152. 10.1093/bioinformatics/bts565 23060610 PMC3516142

[B14] Giang T. N. G. Khai T. V. (2023). Effect of fermentation conditions (yeast content and fermentation time) on the ethanol and bioactive compounds of Ca na (*Elaeocarpus hygrophilus* Kurz) wine by using response surface methodology. *BrewingScience* 76 30–37. 10.23763/BrSc23-01giang

[B15] Gilbert J. A. Jansson J. K. Knight R. (2014). The Earth Microbiome project: successes and aspirations. *BMC Biol.* 12:69. 10.1186/s12915-014-0069-1 25184604 PMC4141107

[B16] González S. S. Barrio E. Gafner J. Querol A. (2006). Natural hybrids from *Saccharomyces cerevisiae*, *Saccharomyces bayanus* and *Saccharomyces kudriavzevii* in wine fermentations. *FEMS Yeast Res.* 6 1221–1234. 10.1111/j.1567-1364.2006.00126.x 17156019

[B17] Hacquard S. Garrido-Oter R. González A. Spaepen S. Ackermann G. Lebeis S. et al. (2015). Microbiota and host nutrition across plant and animal kingdoms. *Cell Host Microbe* 17 603–616. 10.1016/j.chom.2015.04.009 25974302

[B18] Hyatt D. Chen G.-L. LoCascio P. F. Land M. L. Larimer F. W. Hauser L. J. (2010). Prodigal: prokaryotic gene recognition and translation initiation site identification. *BMC Bioinformat.* 11:119. 10.1186/1471-2105-11-119 20211023 PMC2848648

[B19] Jolly N. P. Varela C. Pretorius I. S. (2014). Not your ordinary yeast: non- *Saccharomyces* yeasts in wine production uncovered. *FEMS Yeast Res.* 14 215–237. 10.1111/1567-1364.12111 24164726

[B20] Kelly J. Inglis D. Dowling L. Pickering G. (2022). Impact of *Botrytis cinerea* -infected grapes on quality parameters of red wine made from withered grapes. *Aust. J. Grape Wine Res.* 28 439–449. 10.1111/ajgw.12545

[B21] Knight S. Klaere S. Fedrizzi B. Goddard M. R. (2015). Regional microbial signatures positively correlate with differential wine phenotypes: evidence for a microbial aspect to terroir. *Sci. Rep.* 5:14233. 10.1038/srep14233 26400688 PMC4585847

[B22] Li D. Liu C.-M. Luo R. Sadakane K. Lam T.-W. (2015). MEGAHIT: an ultra-fast single-node solution for large and complex metagenomics assembly via succinct *de Bruijn* graph. *Bioinformatics* 31 1674–1676. 10.1093/bioinformatics/btv033 25609793

[B23] Lisov N. Čakar U. Milenković D. Čebela M. Vuković G. Despotović S. et al. (2023). The influence of cabernet sauvignon ripeness, healthy state and maceration time on wine and fermented pomace phenolic profile. *Fermentation* 9:695. 10.3390/fermentation9070695

[B24] Lopez Pinar A. Rauhut D. Ruehl E. Buettner A. (2017). Effects of bunch rot (*Botrytis cinerea*) and powdery mildew (*Erysiphe necator*) fungal diseases on wine aroma. *Front. Chem.* 5:20. 10.3389/fchem.2017.00020 28401146 PMC5368173

[B25] Love M. I. Huber W. Anders S. (2014). Moderated estimation of fold change and dispersion for RNA-seq data with DESeq2. *Genome Biol.* 15:550. 10.1186/s13059-014-0550-8 25516281 PMC4302049

[B26] Ludlow C. L. Cromie G. A. Garmendia-Torres C. Sirr A. Hays M. Field C. et al. (2016). Independent origins of yeast associated with coffee and cacao fermentation. *Curr. Biol.* 26 965–971. 10.1016/j.cub.2016.02.012 27020745 PMC4821677

[B27] Mallick H. Rahnavard A. McIver L. J. Ma S. Zhang Y. Nguyen L. H. et al. (2021). Multivariable association discovery in population-scale meta-omics studies. *PLoS Comput. Biol.* 17:e1009442. 10.1371/journal.pcbi.1009442 34784344 PMC8714082

[B28] Marchal R. Salmon T. Gonzalez R. Kemp B. Vrigneau C. Williams P. et al. (2020). Impact of *Botrytis cinerea* contamination on the characteristics and foamability of yeast macromolecules released during the alcoholic fermentation of a model grape juice. *Molecules* 25:472. 10.3390/molecules25030472 31979163 PMC7037752

[B29] Mateo J. Maicas S. (2016). Application of non-saccharomyces yeasts to wine-making process. *Fermentation* 2:14. 10.3390/fermentation2030014

[B30] Mezzasalma V. Sandionigi A. Bruni I. Bruno A. Lovicu G. Casiraghi M. et al. (2017). Grape microbiome as a reliable and persistent signature of field origin and environmental conditions in Cannonau wine production. *PLoS One* 12:e0184615. 10.1371/journal.pone.0184615 28892512 PMC5593190

[B31] Morgan H. H. Du Toit M. Setati M. E. (2017). The grapevine and wine microbiome: insights from high-throughput amplicon sequencing. *Front. Microbiol.* 8:820. 10.3389/fmicb.2017.00820 28553266 PMC5425579

[B32] Osborne J. P. Edwards C. G. (2005). Bacteria important during winemaking. *Adv. Food Nutr. Res.* 50 139–177. 10.1016/S1043-4526(05)50005-6 16263430

[B33] Padilla B. Gil J. V. Manzanares P. (2016). Past and future of non-saccharomyces yeasts: from spoilage microorganisms to biotechnological tools for improving wine aroma complexity. *Front. Microbiol.* 7:411. 10.3389/fmicb.2016.00411 27065975 PMC4814449

[B34] Parlapani F. F. Meziti A. Kormas K. Ar Boziaris I. S. (2013). Indigenous and spoilage microbiota of farmed sea bream stored in ice identified by phenotypic and 16S rRNA gene analysis. *Food Microbiol.* 33 85–89. 10.1016/j.fm.2012.09.001 23122505

[B35] Pinto C. Pinho D. Cardoso R. Custódio V. Fernandes J. Sousa S. et al. (2015). Wine fermentation microbiome: a landscape from different Portuguese wine appellations. *Front. Microbiol.* 6:905. 10.3389/fmicb.2015.00905 26388852 PMC4555975

[B36] Segata N. Izard J. Waldron L. Gevers D. Miropolsky L. Garrett W. S. et al. (2011). Metagenomic biomarker discovery and explanation. *Genome Biol.* 12:R60. 10.1186/gb-2011-12-6-r60 21702898 PMC3218848

[B37] Setati M. E. Jacobson D. Bauer F. F. (2015). Sequence-based analysis of the *Vitis vinifera* L. cv cabernet sauvignon grape must mycobiome in three South African vineyards employing distinct agronomic systems. *Front. Microbiol.* 6:1358. 10.3389/fmicb.2015.01358 26648930 PMC4663253

[B38] Stefanini I. Cavalieri D. (2018). Metagenomic approaches to investigate the contribution of the vineyard environment to the quality of wine fermentation: potentials and difficulties. *Front. Microbiol.* 9:991. 10.3389/fmicb.2018.00991 29867889 PMC5964215

[B39] Stefanini I. Dapporto L. Legras J.-L. Calabretta A. Di Paola M. De Filippo C. et al. (2012). Role of social wasps in *Saccharomyces cerevisiae* ecology and evolution. *Proc. Natl. Acad. Sci. U. S. A.* 109 13398–13403. 10.1073/pnas.1208362109 22847440 PMC3421210

[B40] Sumner L. W. Amberg A. Barrett D. Beale M. H. Beger R. Daykin C. A. et al. (2007). Proposed minimum reporting standards for chemical analysis: chemical analysis working group (CAWG) metabolomics standards initiative (MSI). *Metabolomics* 3 211–221. 10.1007/s11306-007-0082-2 24039616 PMC3772505

[B41] Urso R. Rantsiou K. Dolci P. Rolle L. Comi G. Cocolin L. (2008). Yeast biodiversity and dynamics during sweet wine production as determined by molecular methods. *FEMS Yeast Res.* 8 1053–1062. 10.1111/j.1567-1364.2008.00364.x 18341578

[B42] Vejarano R. (2020). Non-saccharomyces in winemaking: source of mannoproteins, nitrogen, enzymes, and antimicrobial compounds. *Fermentation* 6:76. 10.3390/fermentation6030076

[B43] Vilela A. (2018). *Lachancea thermotolerans*, the non-saccharomyces yeast that reduces the volatile acidity of wines. *Fermentation* 4:56. 10.3390/fermentation4030056

[B44] Wang C. Mas A. Esteve-Zarzoso B. (2015). Interaction between *Hanseniaspora uvarum* and *Saccharomyces cerevisiae* during alcoholic fermentation. *Int. J. Food Microbiol.* 206 67–74. 10.1016/j.ijfoodmicro.2015.04.022 25956738

[B45] Zakrzewska A. Van Eikenhorst G. Burggraaff J. E. C. Vis D. J. Hoefsloot H. Delneri D. et al. (2011). Genome-wide analysis of yeast stress survival and tolerance acquisition to analyze the central trade-off between growth rate and cellular robustness. *MBoC* 22 4435–4446. 10.1091/mbc.e10-08-0721 21965291 PMC3216668

[B46] Zarraonaindia I. Owens S. M. Weisenhorn P. West K. Hampton-Marcell J. Lax S. et al. (2015). The soil microbiome influences grapevine-associated microbiota. *Proc. Natl. Acad. Sci. U. S. A.* 112 E139–E148. 10.1128/mBio.02527-14 25805735 PMC4453523

[B47] Zulini L. Fabro E. Peterlunger E. (2005). Characterisation of the grapevine cultivar Picolit by means of morphological descriptors and molecular markers. *Vitis* 44 35–38.

