# Supplementary material for: Multi-omic characterization of microbial dynamics during spontaneous fermentation of sweet wine Picolit variety
Source: Front Microbiol. 2026 Jul 29;17:1857803. doi: 10.3389/fmicb.2026.1857803 (PMC13462049; doi:10.3389/fmicb.2026.1857803)
Supplement: Supplementary file 1 [file Image_2.pdf]

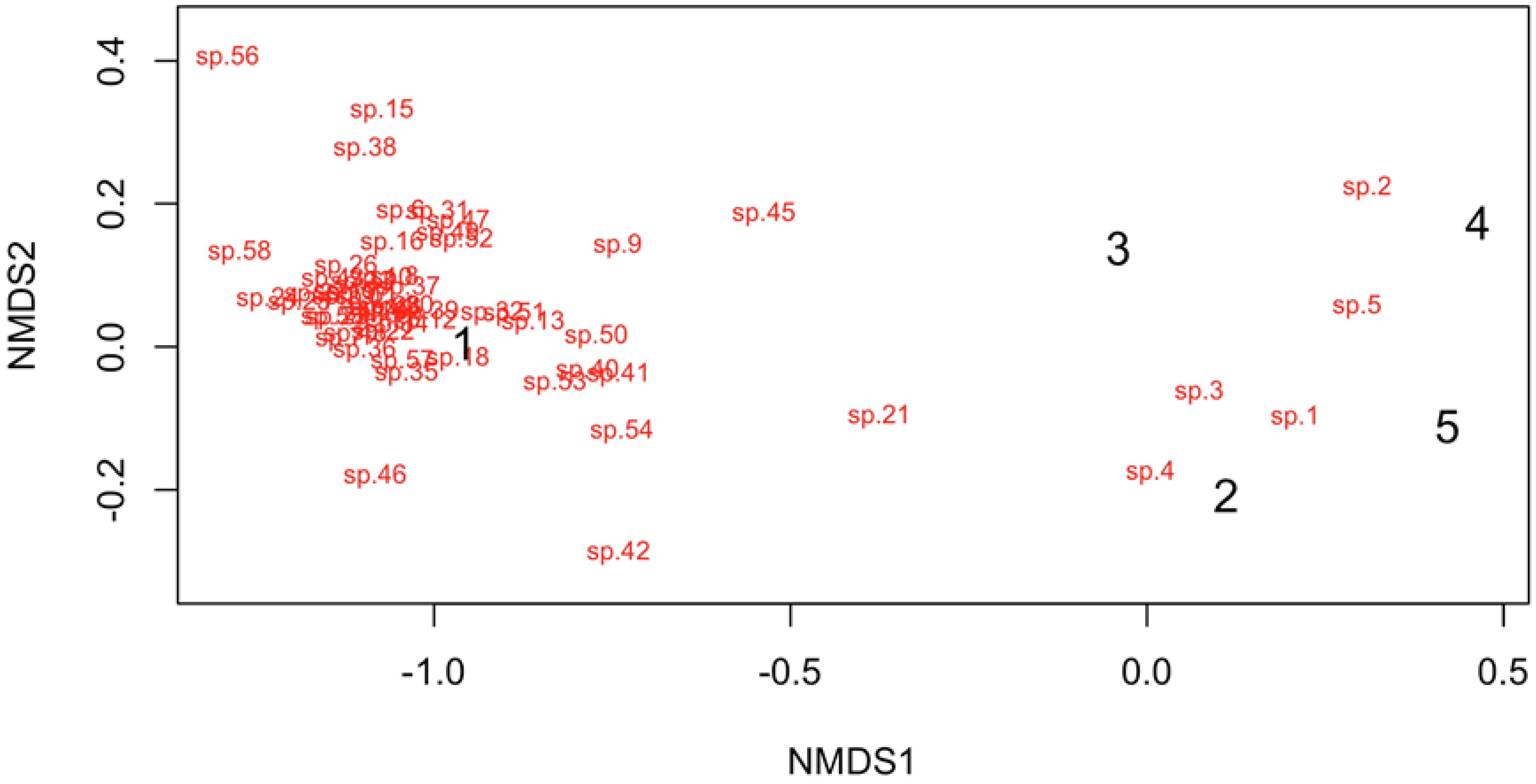

NMDS2
NMDS1
0.4
0.2
0.0
-0.2
-1.0
-0.5
0.0
0.5
sp.56
sp.15
sp.38
sp.45
sp.2
sp.58
sp.9
3
4
sp.16
sp.52
sp.31
sp.47
sp.26
sp.37
sp.13
sp.50
sp.5
1
sp.22
sp.36
sp.18
sp.35
sp.53
sp.41
sp.3
sp.1
5
sp.21
sp.54
sp.4
2
sp.46
sp.42
