# Supplementary material for: Multi-omic characterization of microbial dynamics during spontaneous fermentation of sweet wine Picolit variety
Source: Front Microbiol. 2026 Jul 29;17:1857803. doi: 10.3389/fmicb.2026.1857803 (PMC13462049; doi:10.3389/fmicb.2026.1857803)
Supplement: Supplementary file 2 [file Image_3.pdf]

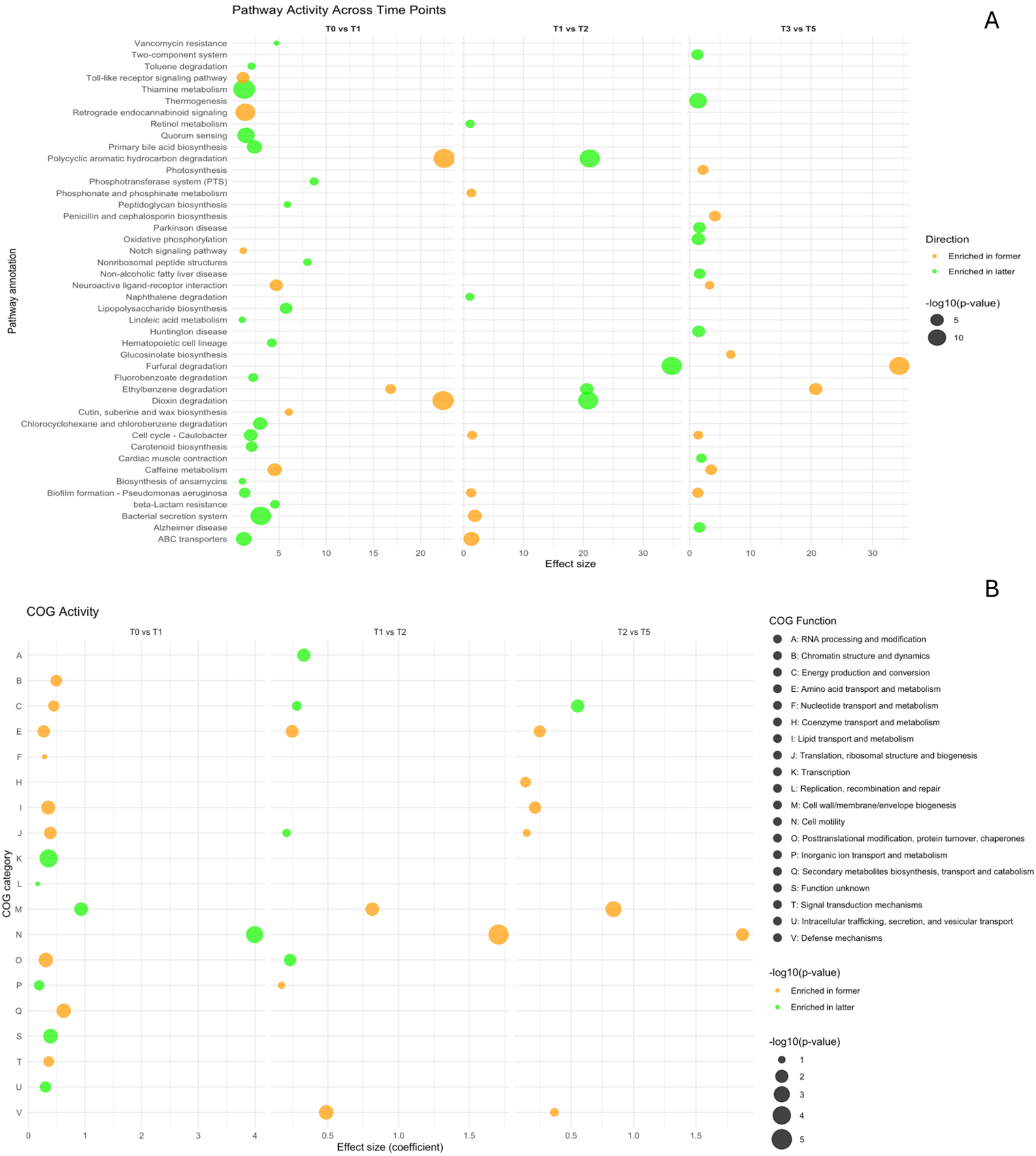
A
Pathway Activity Across Time Points
T0 vs T1
T1 vs T2
T3 vs T5
Pathway annotation
Vancomycin resistance
Two-component system
Toluene degradation
Toll-like receptor signaling pathway
Thiamine metabolism
Thermogenesis
Retrograde endocannabinoid signaling
Retinol metabolism
Quorum sensing
Primary bile acid biosynthesis
Polycyclic aromatic hydrocarbon degradation
Photosynthesis
Phosphotransferase system (PTS)
Phosphonate and phosphinate metabolism
Peptidoglycan biosynthesis
Penicillin and cephalosporin biosynthesis
Parkinson disease
Oxidative phosphorylation
Notch signaling pathway
Nonribosomal peptide structures
Non-alcoholic fatty liver disease
Neuroactive ligand-receptor interaction
Naphthalene degradation
Lipopolysaccharide biosynthesis
Linoleic acid metabolism
Huntington disease
Hematopoietic cell lineage
Glucosinolate biosynthesis
Furfural degradation
Fluorobenzoate degradation
Ethylbenzene degradation
Dioxin degradation
Cutin, suberine and wax biosynthesis
Chlorocyclohexane and chlorobenzene degradation
Cell cycle - Caulobacter
Carotenoid biosynthesis
Cardiac muscle contraction
Caffeine metabolism
Biosynthesis of ansamycins
Biofilm formation - Pseudomonas aeruginosa
beta-Lactam resistance
Bacterial secretion system
Alzheimer disease
ABC transporters
5 10 15 20 0 10 20 30 0 10 20 30
Effect size
Direction
Enriched in former
Enriched in latter
-log10(p-value)
5
10
B
COG Activity
T0 vs T1
T1 vs T2
T2 vs T5
COG category
A B C E F H I J K L M N O P Q S T U V
0 1 2 3 4 0.5 1.0 1.5 0.5 1.0 1.5
Effect size (coefficient)
COG Function
A: RNA processing and modification
B: Chromatin structure and dynamics
C: Energy production and conversion
E: Amino acid transport and metabolism
F: Nucleotide transport and metabolism
H: Coenzyme transport and metabolism
I: Lipid transport and metabolism
J: Translation, ribosomal structure and biogenesis
K: Transcription
L: Replication, recombination and repair
M: Cell wall/membrane/envelope biogenesis
N: Cell motility
O: Posttranslational modification, protein turnover, chaperones
P: Inorganic ion transport and metabolism
Q: Secondary metabolites biosynthesis, transport and catabolism
S: Function unknown
T: Signal transduction mechanisms
U: Intracellular trafficking, secretion, and vesicular transport
V: Defense mechanisms
-log10(p-value)
Enriched in former
Enriched in latter
-log10(p-value)
1
2
3
4
5
