# Supplementary material for: Multi-omic characterization of microbial dynamics during spontaneous fermentation of sweet wine Picolit variety
Source: Front Microbiol. 2026 Jul 29;17:1857803. doi: 10.3389/fmicb.2026.1857803 (PMC13462049; doi:10.3389/fmicb.2026.1857803)
Supplement: Supplementary file 3 [file Image_4.pdf]

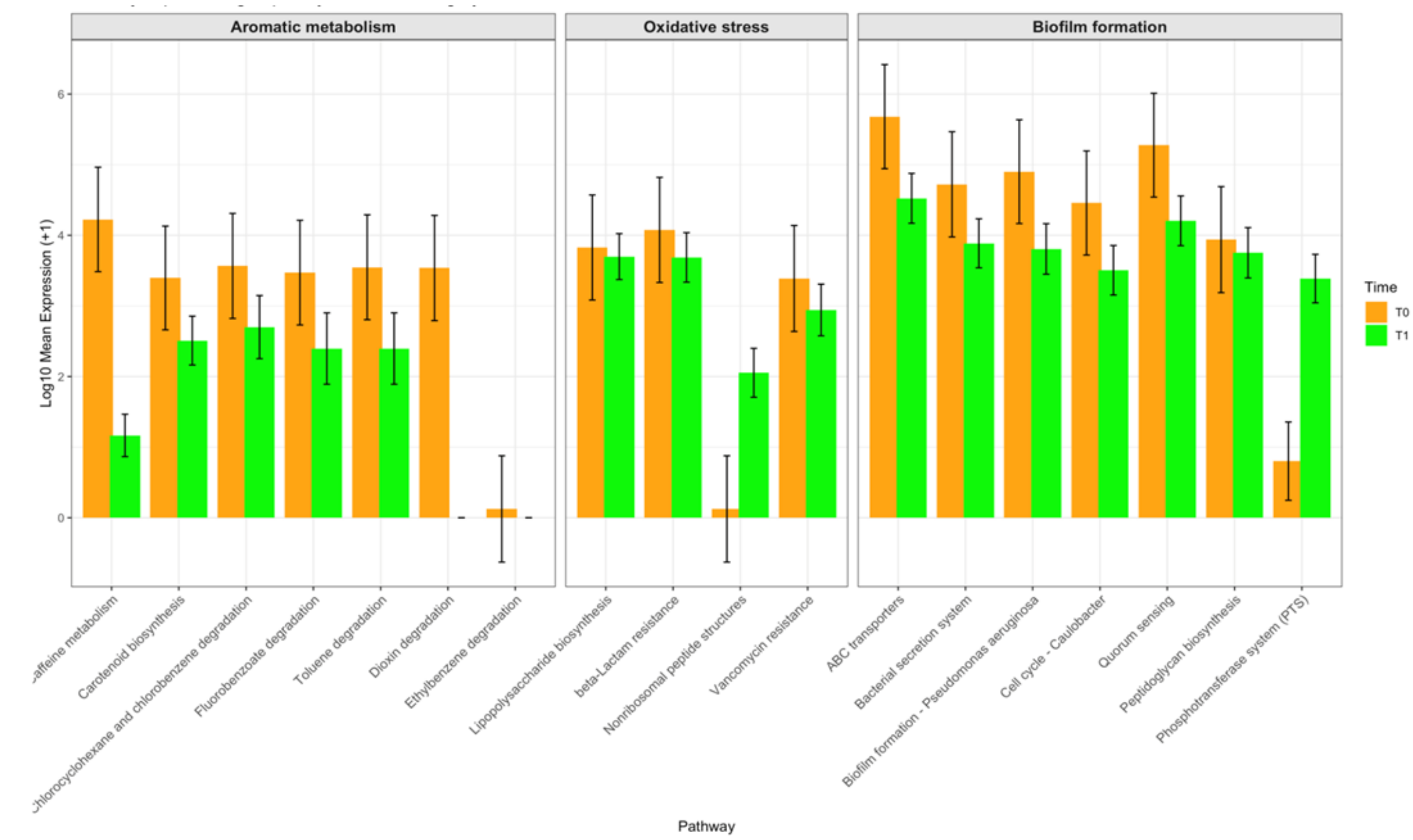

Aromatic metabolism
Oxidative stress
Biofilm formation
Log10 Mean Expression (+1)
0
2
4
6
affeine metabolism
Carotenoid biosynthesis
hlorocyclohexane and chlorobenzene degradation
Fluorobenzoate degradation
Toluene degradation
Dioxin degradation
Ethylbenzene degradation
Lipopolysaccharide biosynthesis
beta-Lactam resistance
Nonribosomal peptide structures
Vancomycin resistance
ABC transporters
Bacterial secretion system
Biofilm formation - Pseudomonas aeruginosa
Cell cycle - Caulobacter
Quorum sensing
Peptidoglycan biosynthesis
Phosphotransferase system (PTS)
Pathway
Time
T0
T1

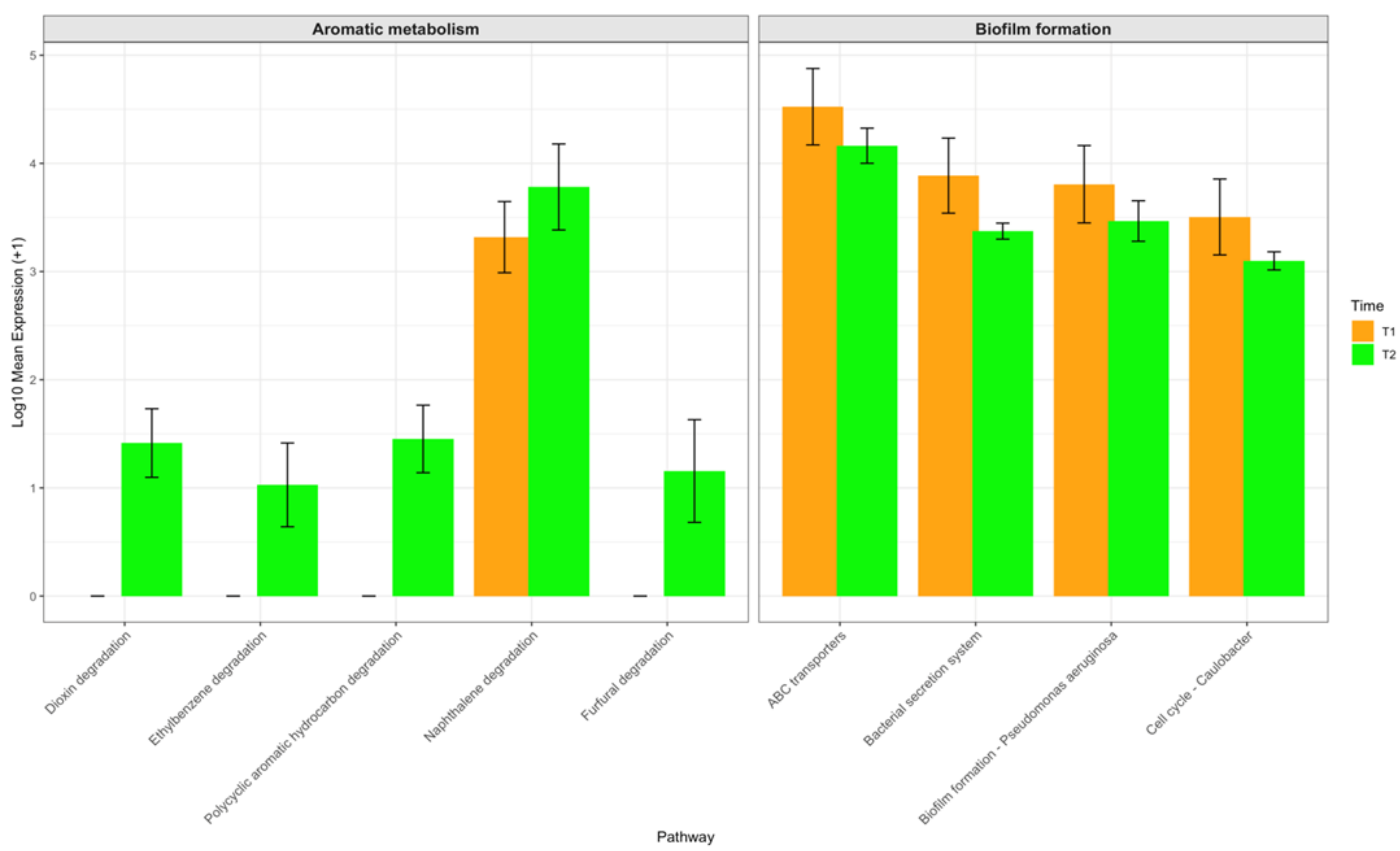

Aromatic metabolism
Biofilm formation
Log10 Mean Expression (+1)
0
1
2
3
4
5
Dioxin degradation
Ethylbenzene degradation
Polycyclic aromatic hydrocarbon degradation
Naphthalene degradation
Furfural degradation
ABC transporters
Bacterial secretion system
Biofilm formation - Pseudomonas aeruginosa
Cell cycle - Caulobacter
Pathway
Time
T1
T2

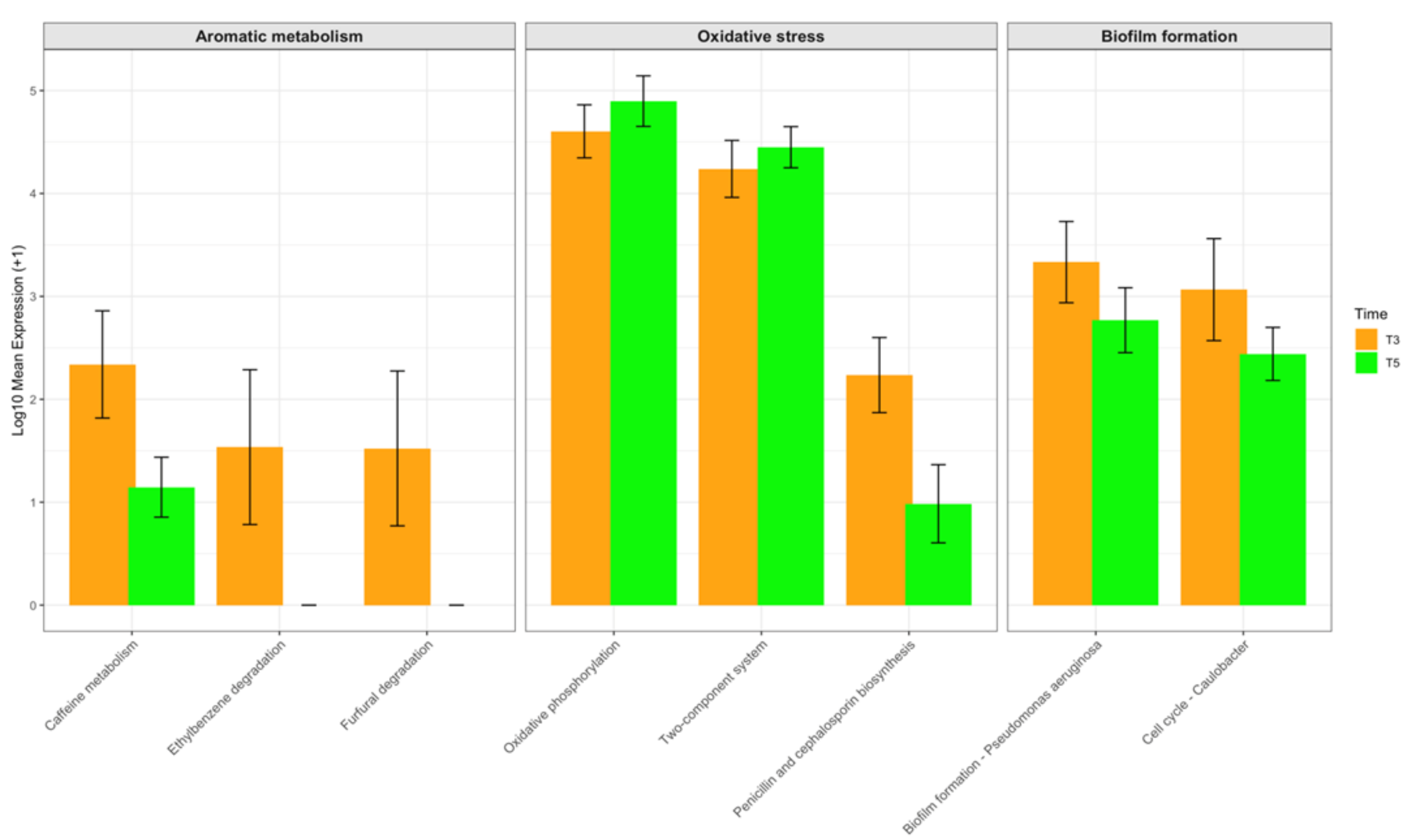

Aromatic metabolism
Oxidative stress
Biofilm formation
Log10 Mean Expression (+1)
0
1
2
3
4
5
Caffeine metabolism
Ethylbenzene degradation
Furfural degradation
Oxidative phosphorylation
Two-component system
Penicillin and cephalosporin biosynthesis
Biofilm formation - Pseudomonas aeruginosa
Cell cycle - Caulobacter
Pathway
Time
T3
T5
