# Supplementary material for: Multi-omic characterization of microbial dynamics during spontaneous fermentation of sweet wine Picolit variety
Source: Front Microbiol. 2026 Jul 29;17:1857803. doi: 10.3389/fmicb.2026.1857803 (PMC13462049; doi:10.3389/fmicb.2026.1857803)
Supplement: Supplementary file 4 [file Image_6.pdf]

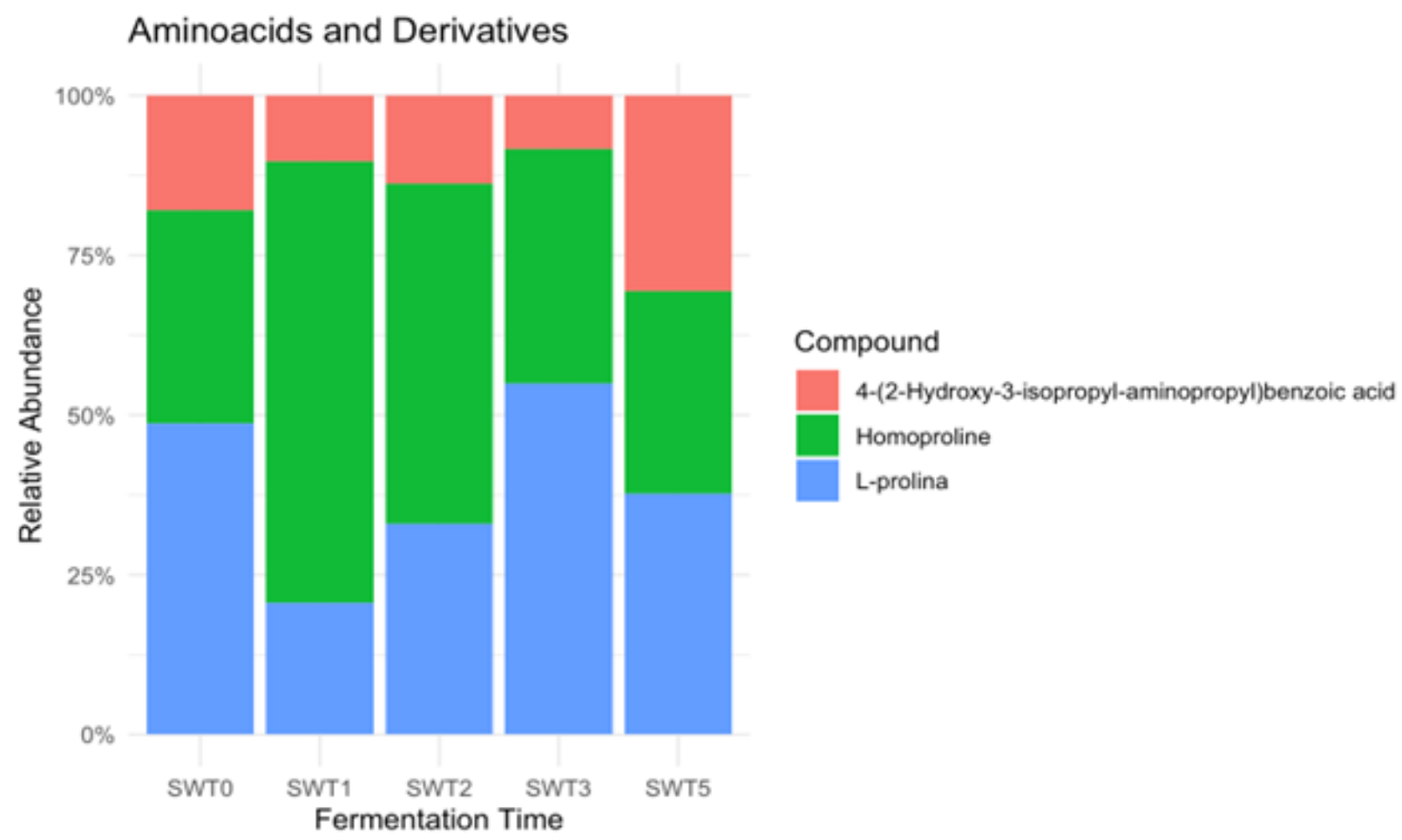
Aminoacids and Derivatives
Relative Abundance
100%
75%
50%
25%
0%
SWT0
SWT1
SWT2
SWT3
SWT5
Fermentation Time
Compound
4-(2-Hydroxy-3-isopropyl-aminopropyl)benzoic acid
Homoproline
L-prolina

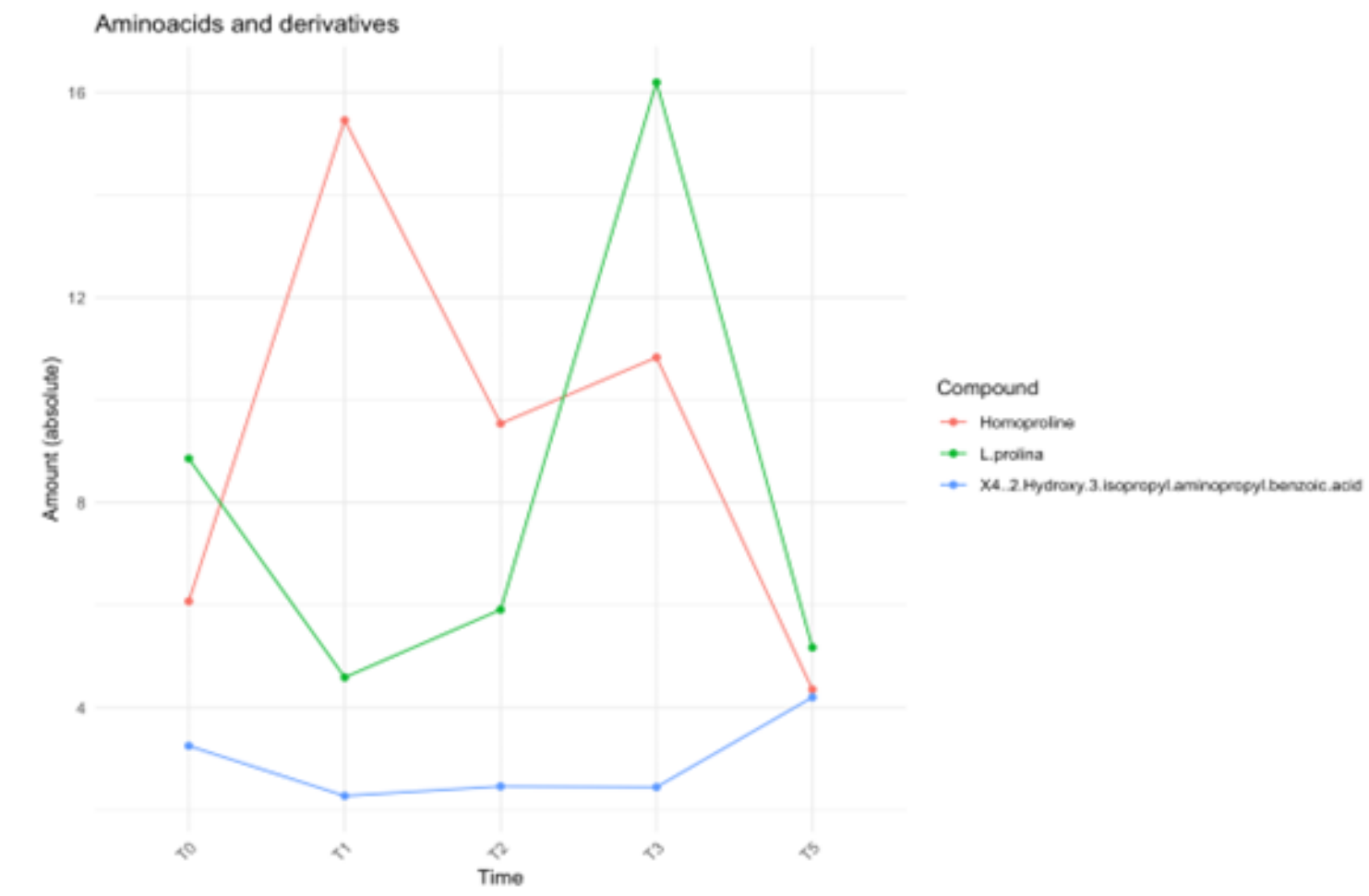
Aminoacids and derivatives
Amount (absolute)
Time
Compound
Homoproline
L.prolina
X4..2.Hydroxy.3.isopropyl.aminopropyl.benzoic.acid

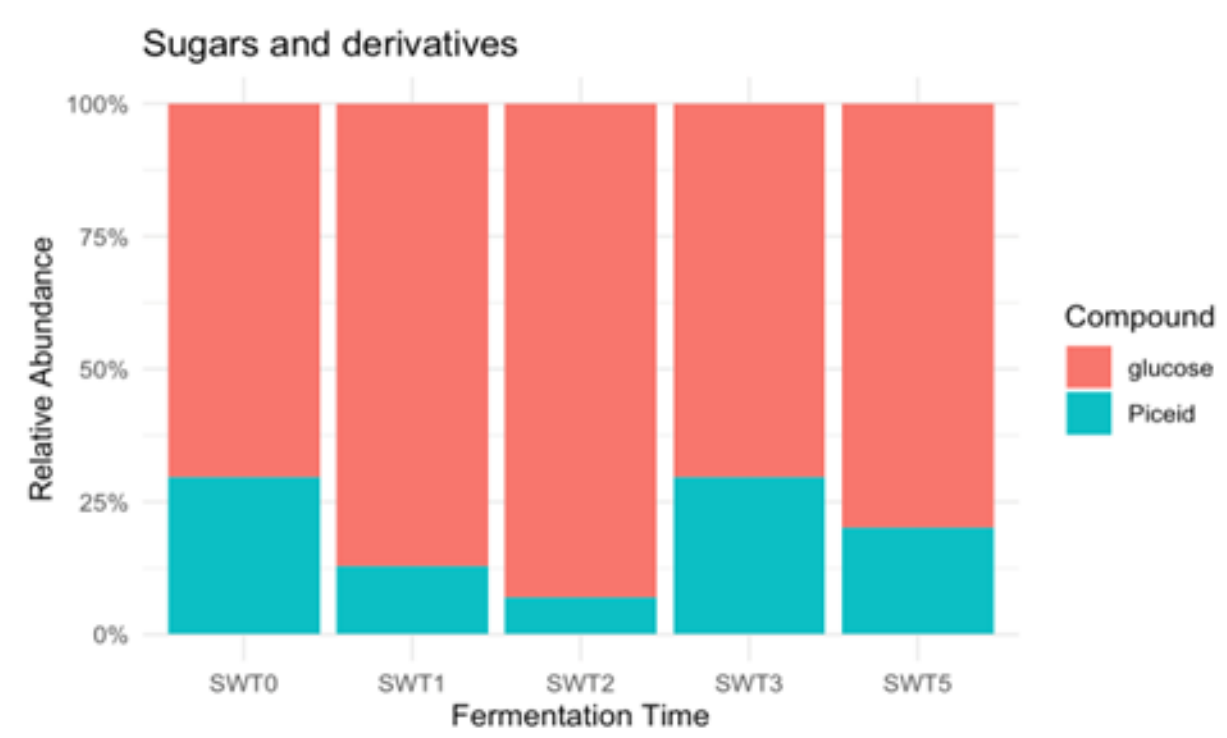
Sugars and derivatives
Relative Abundance
100%
75%
50%
25%
0%
SWT0
SWT1
SWT2
SWT3
SWT5
Fermentation Time
Compound
glucose
Piceid

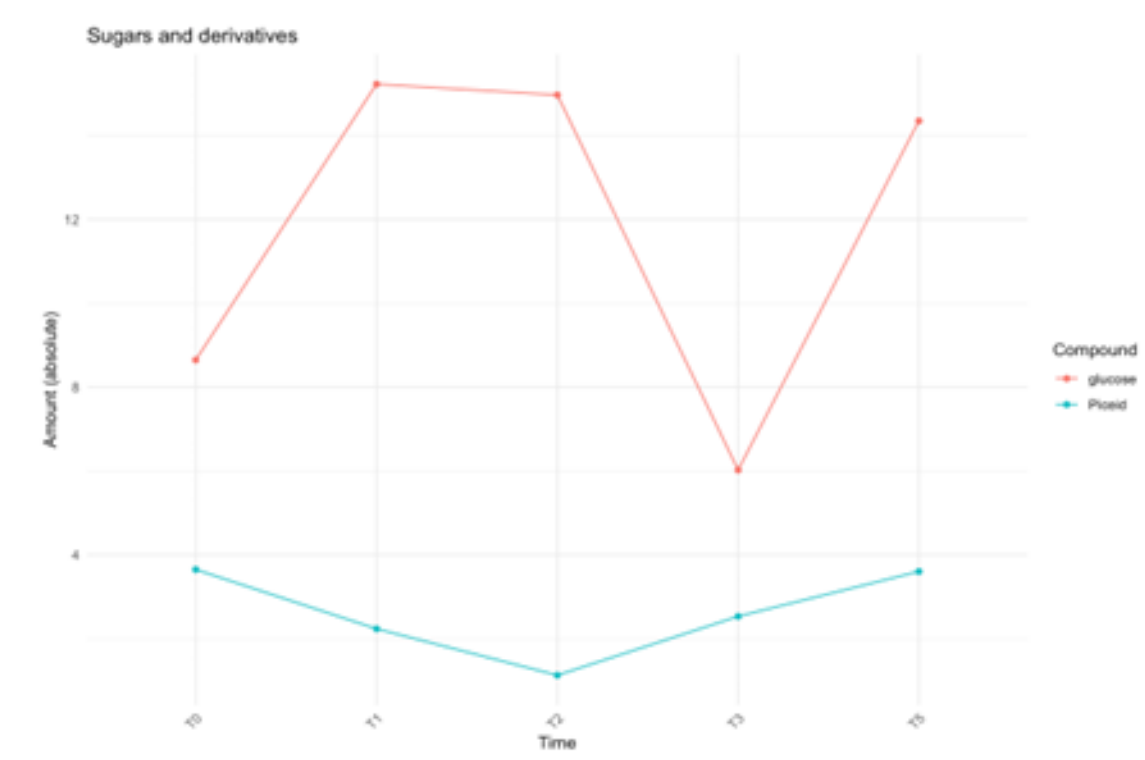
Sugars and derivatives
Amount (absolute)
Time

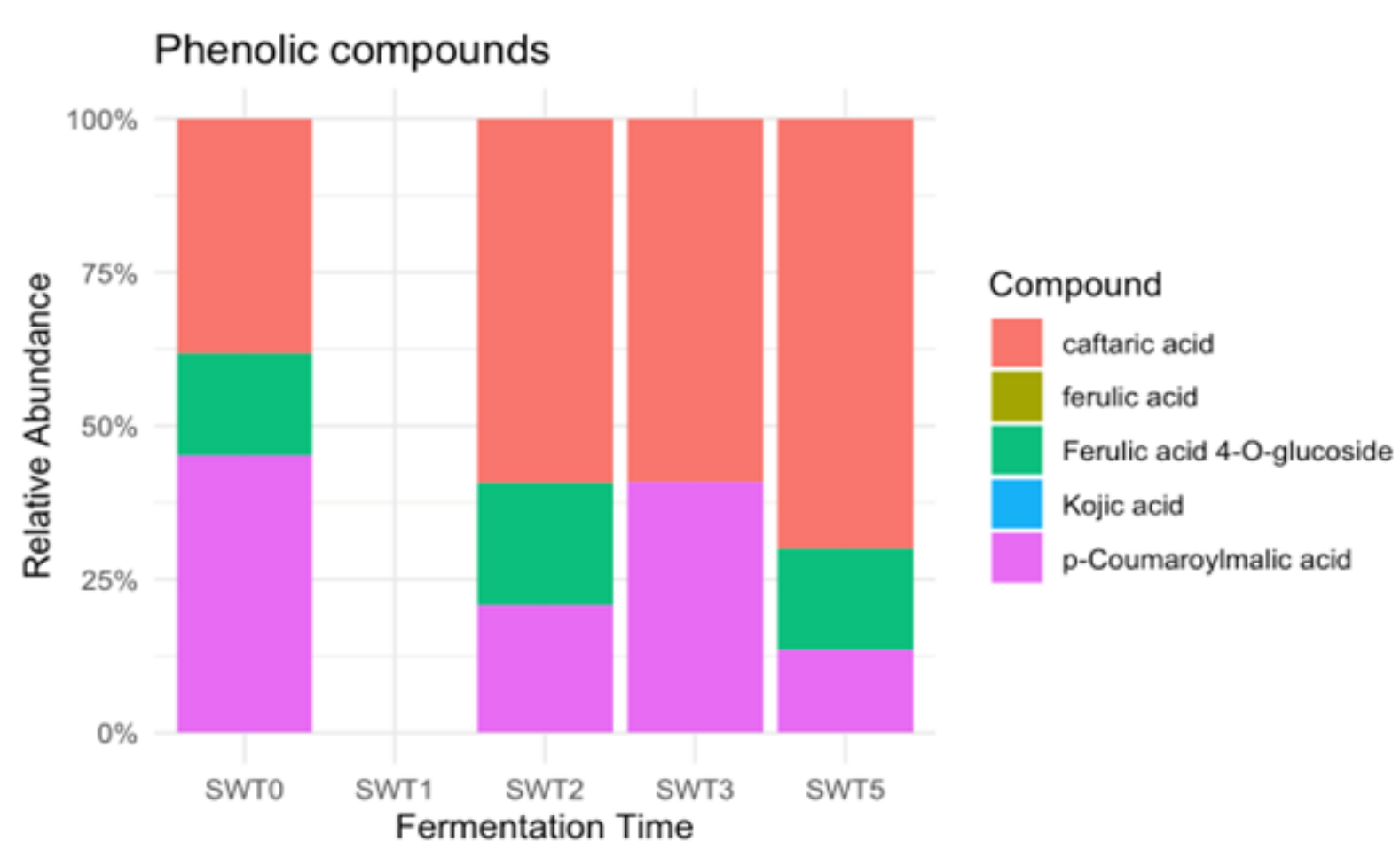
Phenolic compounds
Relative Abundance
100%
75%
50%
25%
0%
SWT0
SWT1
SWT2
SWT3
SWT5
Fermentation Time
Compound
caftaric acid
ferulic acid
Ferulic acid 4-O-glucoside
Kojic acid
p-Coumaroylmalic acid

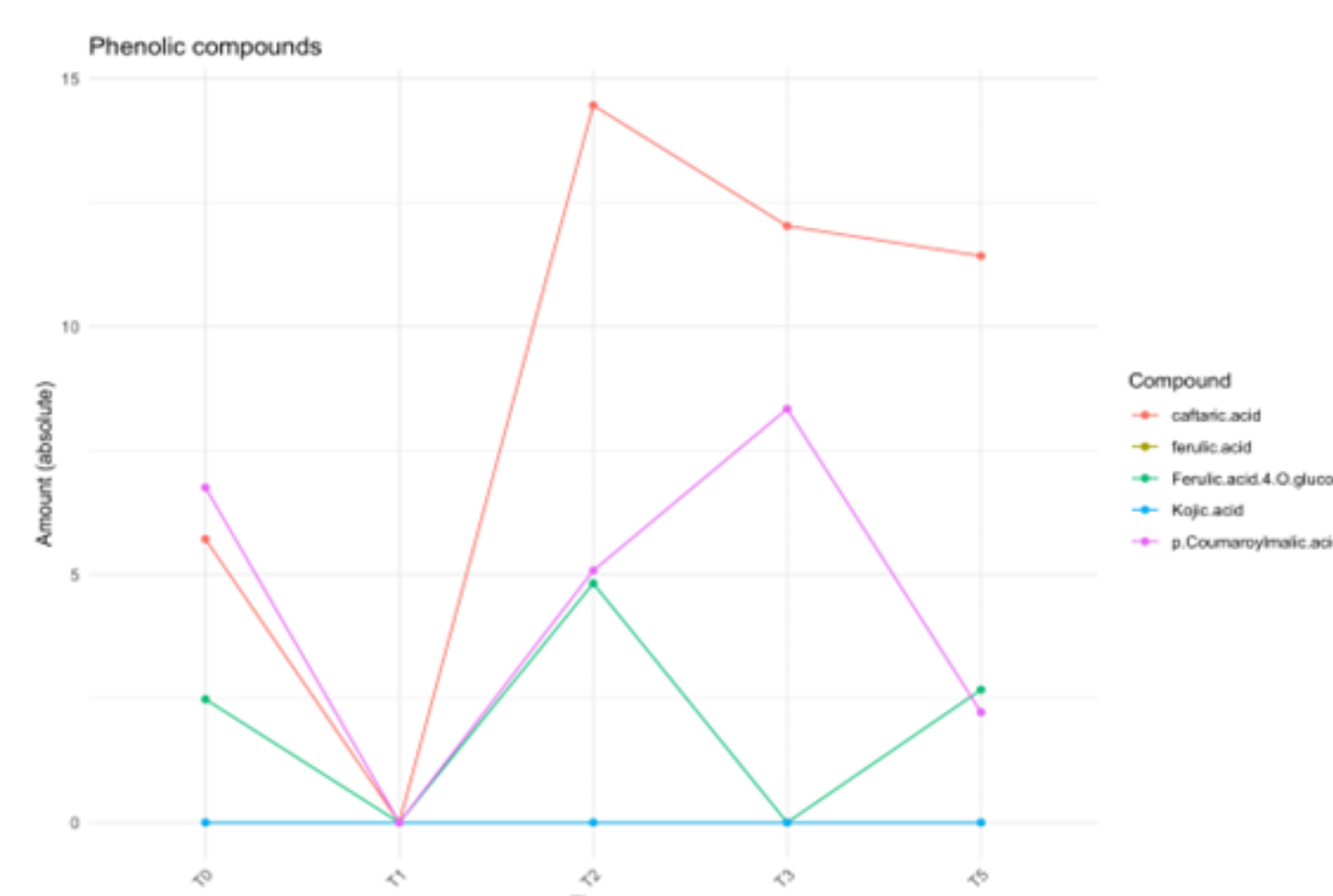
Phenolic compounds
Amount (absolute)
15
10
5
0
Time
Compound
caftaric.acid
ferulic.acid
Ferulic.acid.4.O.glucoside
Kojic.acid
p.Coumaroylmalic.acid

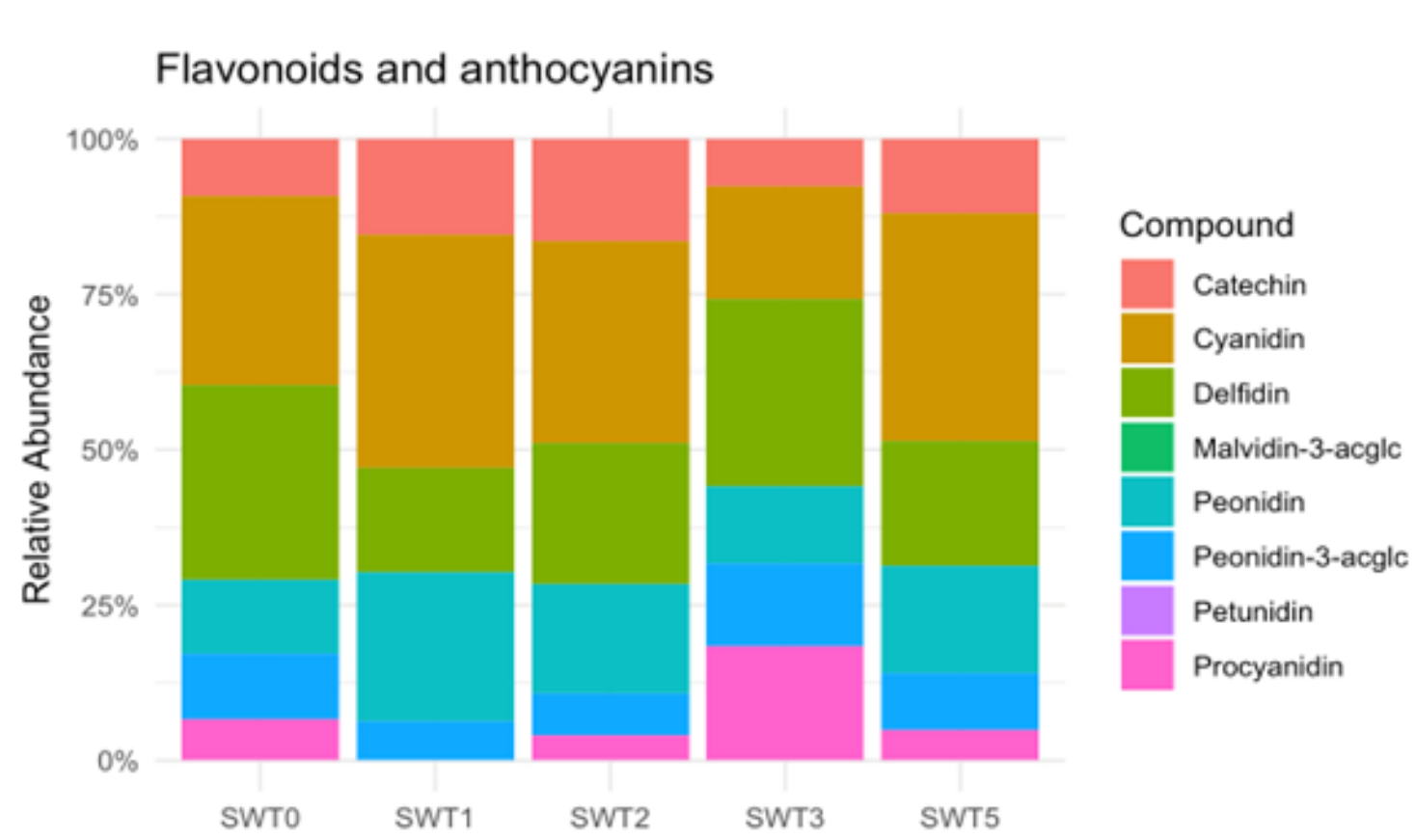
Flavonoids and anthocyanins
Relative Abundance
100%
75%
50%
25%
0%
SWT0
SWT1
SWT2
SWT3
SWT5
Fermentation Time
Compound
Catechin
Cyanidin
Delfidin
Malvidin-3-acglc
Peonidin
Peonidin-3-acglc
Petunidin
Procyanidin

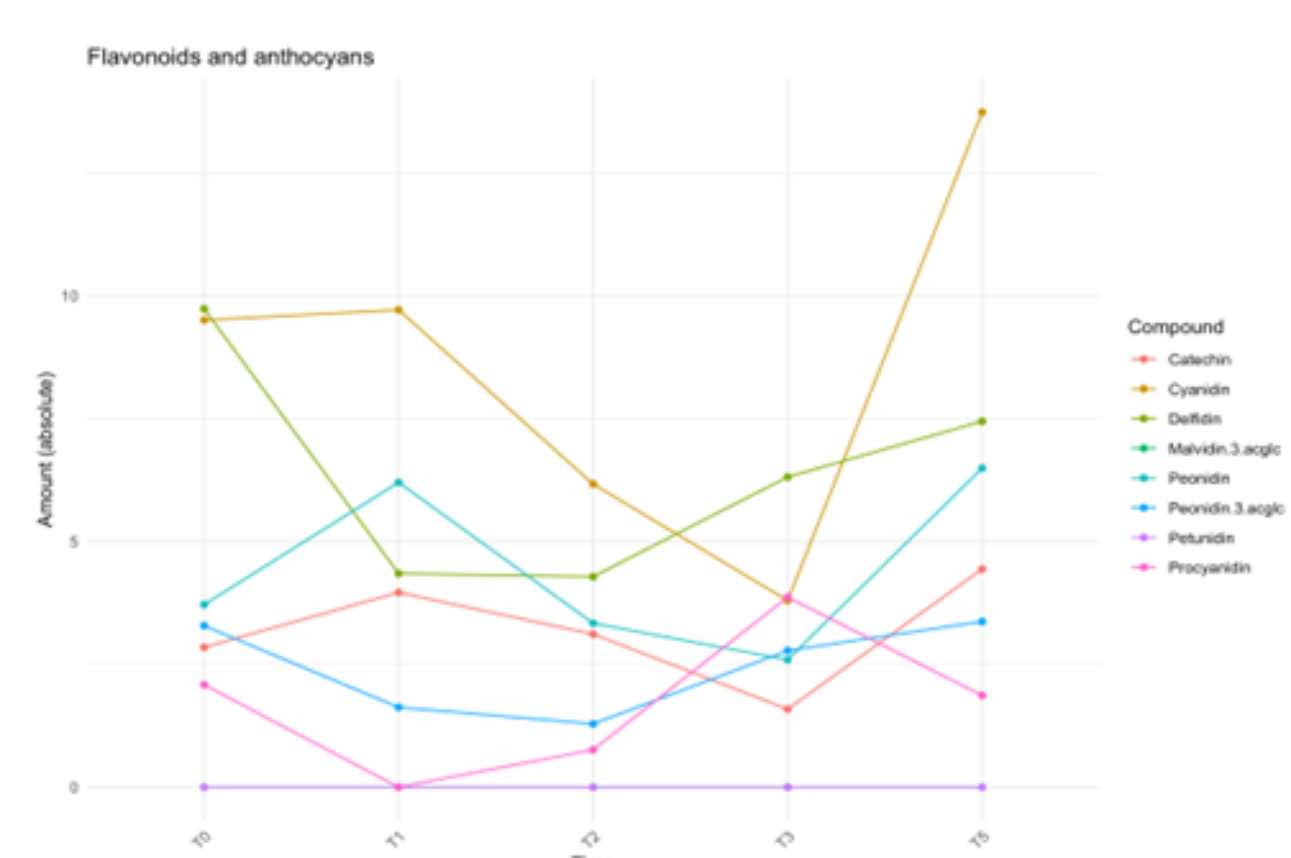
Flavonoids and anthocyans
Amount (absolute)
Time
Compound
Catechin
Cyanidin
Delfidin
Malvidin.3.acglc
Peonidin
Peonidin.3.acglc
Petunidin
Procyanidin

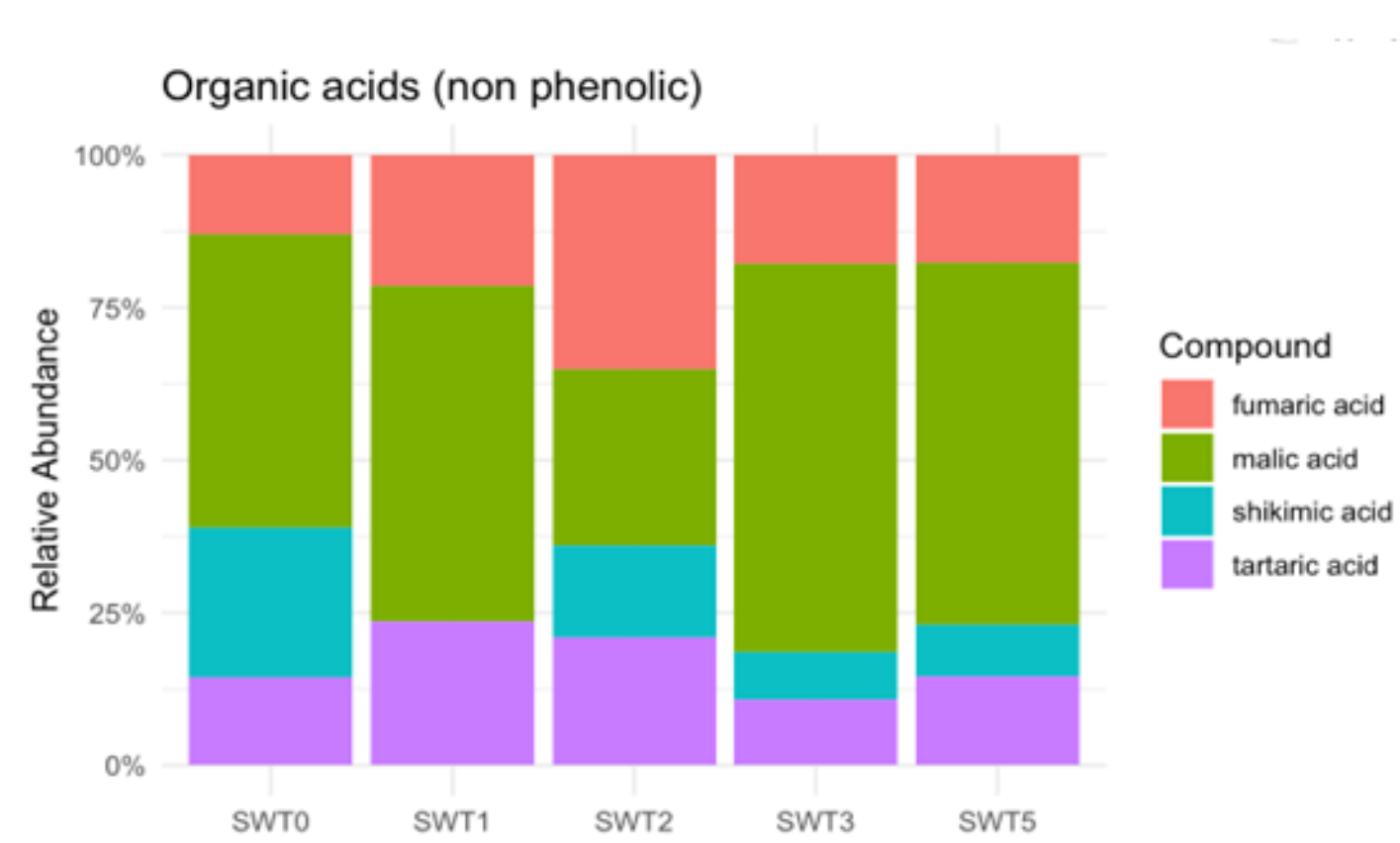
Organic acids (non phenolic)
Relative Abundance
100%
75%
50%
25%
0%
SWT0
SWT1
SWT2
SWT3
SWT5
Fermentation Time (h)
Compound
fumaric acid
malic acid
shikimic acid
tartaric acid

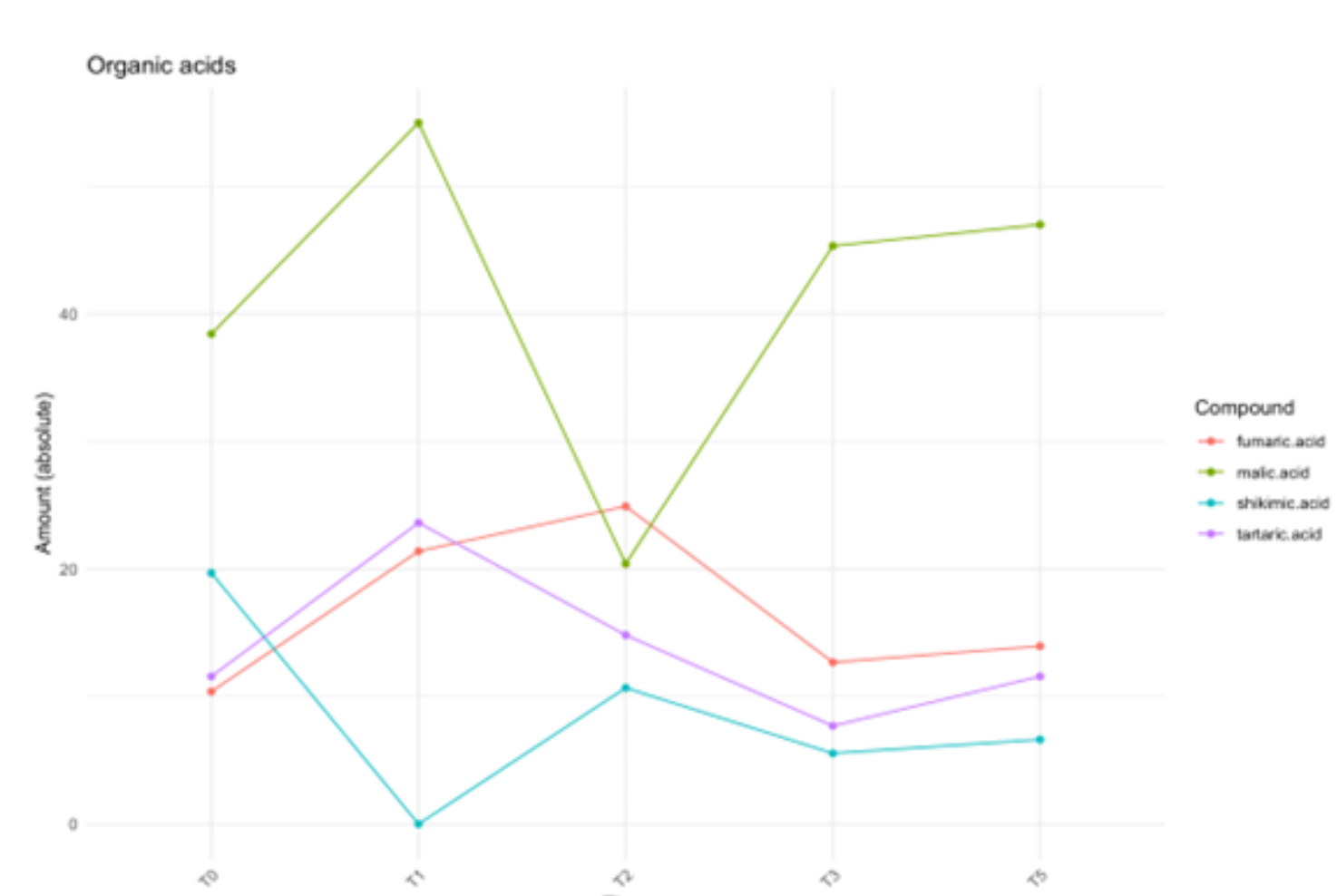
Organic acids
Amount (absolute)
40
20
0
Time
Compound
fumaric.acid
malic.acid
shikimic.acid
tartaric.acid
