# Supplementary material for: Multi-omic characterization of microbial dynamics during spontaneous fermentation of sweet wine Picolit variety
Source: Front Microbiol. 2026 Jul 29;17:1857803. doi: 10.3389/fmicb.2026.1857803 (PMC13462049; doi:10.3389/fmicb.2026.1857803)
Supplement: Supplementary file 5 [file Data_Sheet_1.docx]

**Figure S1. Histogram of distribution of taxon.**

(A) Abundance-based filtering threshold for taxa inclusion. Histogram displaying the log10-transformed abundance distribution of all taxa prior to filtering. The vertical dashed blue line denotes the minimum abundance threshold applied to remove extremely low-count taxa that may derive from sequencing noise or sporadic detection. Taxa to the left of the threshold are excluded from downstream analyses, whereas those to the right are retained. (B) Prevalence-based filtering threshold for taxa inclusion. Histogram showing how many samples contain each taxon. The dashed blue line marks the prevalence cutoff. Taxa detected in fewer samples than this threshold are removed to ensure statistical robustness and minimize false positives driven by ultra-rare features. The retained taxa represent a more reliable subset of the microbial community.

**Figure S2. NMDS ordination of fungal and bacterial communities during spontaneous fermentation**
NMDS ordination based on Bray–Curtis dissimilarities of the fungal community detected across fermentation stages. Labels “sp.n” indicate individual species ordination identifiers, where n corresponds to the species number. Fermentation stages are indicated by numbers 0–5, corresponding to the sampled fermentation time points (T0–T5).

**Figure S3. Differential pathway (KEGG) and functional (COG) analyses across fermentation stages**

Panel A presents a bubble plot of KEGG pathways identified as differentially abundant between consecutive fermentation stages; each point represents a pathway, with color indicating the stage in which it is enriched and bubble size reflecting the corresponding LDA effect size.
Panel B shows a bubble plot of differentially abundant COG functional categories derived from MaAsLin2 models; each point represents a COG category, positioned along the x-axis according to the model coefficient, with bubble size indicating statistical significance. Only pathways and categories meeting the predefined significance thresholds are reported.

**Figure S4. Pairwise comparisons of pathway-level functional potential across fermentation stages**

The figure reports KEGG pathway abundances for each pairwise comparison between fermentation stages (T0–T1, T1–T2, T3–T5). Bar plots display the log₁₀-transformed read counts associated with each pathway, grouped by functional category (aromatic metabolism, oxidative stress, biofilm formation). Error bars indicate variation among replicates. Pathways enriched in the earlier or later stage of each comparison are shown according to the direction of differential abundance.

**Figure S5. Correlation networks between microbial taxa and metabolic pathways**

Network graphs illustrating significant Spearman correlations between microbial taxa and KEGG pathways across fermentation stages. (A) the figure shows correlations for fungal taxa; nodes represent fungal genera or species and metabolic pathways, with edges indicating significant positive or negative associations. (B) the figure displays the corresponding bacterial correlation network, using the same node and edge conventions. Only correlations meeting the predefined statistical thresholds are included.

**Figure S6. Temporal variation of individual metabolite groups across fermentation stages**

Line plots and stacked bar plots illustrating the temporal patterns of major metabolite groups across fermentation stages. Each panel reports, for a specific compound class, the relative abundances (stacked bar plots) and absolute abundances (line plots) of individual metabolites:
amino acids and derivatives; sugars and their derivatives; phenolic compounds; flavonoids and anthocyanins; non-phenolic organic acids. Each plot depicts metabolite-level trajectories from T0 to T5, based on summed or individual abundance values within each compound class.

**Figure S7. Multi-layer microbial–metabolite–pathway networks across fermentation stages**

Network diagrams showing significant associations among microbial taxa, metabolites, and KEGG pathways at different fermentation stages. Panel displays the main network modules identified at T0, T1, T2, and T3, highlighting the stage-specific organization of microbial, metabolic, and functional interactions. With line red positive correlation, in blue the negative correlation.

**Table S1**. Sequencing depth among replicates

| Sample_ID | Time | Replicates | paired-end | Raw reads | Cleaned reads |
| --- | --- | --- | --- | --- | --- |
| SWT0R1 | T0 | R1 | R1 | 16546093 | 14534796 |
| SWT0R1 | T0 | R1 | R2 | 28812343 | 26246182 |
| SWT0R2 | T0 | R2 | R1 | 16546093 | 14968741 |
| SWT0R2 | T0 | R2 | R2 | 13665026 | 11994705 |
| SWT0R3 | T0 | R3 | R1 | 28812343 | 25485970 |
| SWT0R3 | T0 | R3 | R2 | 13665026 | 12342107 |
| SWT1R1 | T1 | R1 | R1 | 436292 | 315028 |
| SWT1R1 | T1 | R1 | R2 | 57044757 | 42298782 |
| SWT1R2 | T1 | R2 | R1 | 436292 | 350772 |
| SWT1R2 | T1 | R2 | R2 | 49505964 | 35815344 |
| SWT1R3 | T1 | R3 | R1 | 57044757 | 40769622 |
| SWT1R3 | T1 | R3 | R2 | 49505964 | 37176950 |
| SWT2R1 | T2 | R1 | R1 | 54437504 | 39703803 |
| SWT2R1 | T2 | R1 | R2 | 18676852 | 14192757 |
| SWT2R2 | T2 | R2 | R1 | 54437504 | 41211352 |
| SWT2R2 | T2 | R2 | R2 | 27880778 | 19744706 |
| SWT2R3 | T2 | R3 | R1 | 18676852 | 13564452 |
| SWT2R3 | T2 | R3 | R2 | 27880778 | 20539700 |
| SWT3R1 | T3 | R1 | R1 | 26664342 | 22102696 |
| SWT3R1 | T3 | R1 | R2 | 39033389 | 33372120 |
| SWT3R2 | T3 | R2 | R1 | 26664342 | 22745664 |
| SWT3R2 | T3 | R2 | R2 | 1203149 | 797706 |
| SWT3R3 | T3 | R3 | R1 | 39033389 | 32195696 |
| SWT3R3 | T3 | R3 | R2 | 1203149 | 904194 |
| SWT5R1 | T5 | R1 | R1 | 27140031 | 21158213 |
| SWT5R1 | T5 | R1 | R2 | 13616611 | 11209718 |
| SWT5R2 | T5 | R2 | R1 | 27140031 | 21988158 |
| SWT5R2 | T5 | R2 | R2 | 925339 | 685413 |
| SWT5R3 | T5 | R3 | R1 | 13616611 | 10760704 |
| SWT5R3 | T5 | R3 | R2 | 925339 | 768494 |
